# Identification of the novel FOXP3-dependent T_reg_ cell transcription factor MEOX1 by high-dimensional analysis of human CD4^+^ T cells

**DOI:** 10.3389/fimmu.2023.1107397

**Published:** 2023-07-25

**Authors:** Kevin Baßler, Lisa Schmidleithner, Mehrnoush Hadaddzadeh Shakiba, Tarek Elmzzahi, Maren Köhne, Stefan Floess, Rebekka Scholz, Naganari Ohkura, Timothy Sadlon, Kathrin Klee, Anna Neubauer, Shimon Sakaguchi, Simon C. Barry, Jochen Huehn, Lorenzo Bonaguro, Thomas Ulas, Marc Beyer

**Affiliations:** ^1^ Systems Medicine, German Center for Neurodegenerative Diseases (DZNE), Bonn, Germany; ^2^ LIMES-Institute, Laboratory for Genomics and Immunoregulation, University of Bonn, Bonn, Germany; ^3^ Immunogenomics & Neurodegeneration, German Center for Neurodegenerative Diseases (DZNE), Bonn, Germany; ^4^ Department of Microbiology and Immunology, The Peter Doherty Institute for Infection and Immunity, University of Melbourne, Melbourne, VIC, Australia; ^5^ Experimental Immunology, Helmholtz Centre for Infection Research, Braunschweig, Germany; ^6^ Laboratory of Experimental Immunology, WPI Immunology Frontier Research Center, Osaka University, Osaka, Japan; ^7^ Molecular Immunology, Robinson Research Institute, University of Adelaide, Norwich Centre, North Adelaide, SA, Australia; ^8^ PRECISE, Platform for Single Cell Genomics and Epigenomics at the German Center for Neurodegenerative Diseases and the University of Bonn, Bonn, Germany

**Keywords:** Treg cells, MEOX1, Foxp3, regulatory T cells, human CD4

## Abstract

CD4^+^ T cells play a central role in the adaptive immune response through their capacity to activate, support and control other immune cells. Although these cells have become the focus of intense research, a comprehensive understanding of the underlying regulatory networks that orchestrate CD4^+^ T cell function and activation is still incomplete. Here, we analyzed a large transcriptomic dataset consisting of 48 different human CD4^+^ T cell conditions. By performing reverse network engineering, we identified six common denominators of CD4^+^ T cell functionality (CREB1, E2F3, AHR, STAT1, NFAT5 and NFATC3). Moreover, we also analyzed condition-specific genes which led us to the identification of the transcription factor MEOX1 in T_reg_ cells. Expression of MEOX1 was comparable to FOXP3 in T_reg_ cells and can be upregulated by IL-2. Epigenetic analyses revealed a permissive epigenetic landscape for MEOX1 solely in T_reg_ cells. Knockdown of MEOX1 in T_reg_ cells revealed a profound impact on downstream gene expression programs and T_reg_ cell suppressive capacity. These findings in the context of CD4^+^ T cells contribute to a better understanding of the transcriptional networks and biological mechanisms controlling CD4^+^ T cell functionality, which opens new avenues for future therapeutic strategies.

## 1 Introduction

CD4^+^ helper T cells (T_H_ cells) are critically involved in most adaptive immune responses as they are responsible for activation of B cells, enhancing the response of cytotoxic T cells, promoting macrophage function and enabling them to mount an immune response against invading microorganisms (1). T_H_ cells can be divided into different subgroups depending on their function and cytokine production. For example, T_H_1 cells secrete mainly IFN-γ and are important for the defense against intracellular pathogens, while T_H_2 cells produce a variety of cytokines, including IL-4, IL-5, and IL-13, and are involved in mounting an immune response against extracellular parasites (2). T_H_17 cells, on the other hand, produce primarily IL-17 and defend the organism against extracellular bacteria and fungi (3).

However, T_H_ cells are not only involved in the induction of the immune response but also play a vital role in regulation, modulation, and fine-tuning of immune responses through interactions with regulatory T cells (T_reg_ cells). T_reg_ cells exert their regulatory function through a variety of different mechanisms such as expression of inhibitory cytokines or surface markers, direct cytotoxicity or disruption of the metabolism of target cells (4, 5). T_reg_ cells, like all helper T cell subtypes, are dependent on complex interactions of signaling pathways converging in the activity of different transcription factors (6). The major transcription factor responsible for the induction of T_reg_ cell programing is Forkhead box protein 3 (FOXP3), which is involved in the generation, maintenance and function of T_reg_ cells (4, 5). However, even though our understanding of T_reg_ cell biology has greatly improved since their existence was first hypothesized in the 1970s, we still do not completely understand the underlying regulatory networks which mediate T_reg_ cell functionality (7). Modern Omics-technologies in combination with innovative bioinformatic analysis approaches have made it possible to analyze immune cells in more detail and to better understand the underlying mechanisms of the activation, functionality and polarization of different immune populations (8). For myeloid cells, transcriptome analysis of differently stimulated macrophages revolutionized our understanding of different macrophage polarization states (9). We hypothesized that utilizing a similar strategy by combining different complementary bioinformatic analysis approaches would enable us to better understand the orchestration of transcriptional and epigenetic events governing T_reg_ cell programing and provide new insights into T_reg_ cell biology. The usefulness of such approaches has also been documented for T cell biology as bioinformatic analyses have successfully been applied to advance our understanding of T cell differentiation. For instance, we and others identified novel genes which are important for the differentiation and functionality of T_reg_ cells (10–12), T_H_17 cells (13, 14), and T_H_1 cells and T_H_2 cells (15).

The advent of single-cell transcriptomics over the last years has even further increased our appreciation that these transcriptional changes can be governed through the interaction of transcription factors, posttranscriptional changes induced by miRNAs and lncRNAs but also through epigenetic mechanisms including DNA methylation, chromatin accessibility and histone modifications. Previous epigenetic analyses have shown that a better understanding of the underlying cell type specific epigenetic events is important to better understand T cell biology. E.g. nucleosomes around loci of the different lineage-defining T_H_-cell cytokines are differentially methylated between the different T_H_-cell subgroups, while the histone methylation of lineage defining transcription factors is more plastic (16, 17). In addition, DNA methylation has been described as a key element for T cell differentiation with the FOXP3 locus serving as the prime example for DNA methylation being important for T_reg_ cell generation and stability (18).

In light of this, it is obvious that a multi-layered approach analyzing pre-existing or novel datasets has the potential to yield new insights into the mechanisms governing T cell proliferation, differentiation and function and thus may also shed light on different disease progresses. However, validating these findings experimentally can be challenging (11, 19).

In this study, we combined different bioinformatic analysis methods using a systems immunology approach to analyze previously published datasets from human CD4^+^ T_H_ cells including differently stimulated CD4^+^ T_H_ cells and T_reg_ cells to establish common traits for all CD4^+^ T_H_ cells but also to identify new T_reg_ cell signature genes.

Using this approach, we identified the transcription factor MEOX1 (Mesenchyme Homeobox 1) to be highly expressed particularly in activated T_reg_ cells with a similar expression pattern to FOXP3. MEOX1 has been primarily implicated in early development, where MEOX1 is necessary in all somatic compartments to ensure proper development (20), with expression levels dropping with gestational age. Frameshift mutations resulting in an unstable MEOX1 transcript or nonsense mutations of MEOX1 have been described to cause Klippel-Feil-Syndrome, a segmentation defect in the cervical spine (20, 21). Furthermore, MEOX1 has been implicated in the development and progression of breast and non-small cell lung cancer (22, 23). As such, MEOX1 expression has been correlated with breast cancer stage and poor survival (22). However, despite the importance of MEOX1 in both development and cancer, only recently a first report has indicated MEOX1 to be important for T_reg_ cells in the context of the tumor environment in intrahepatic cholangiocarcinoma and the acquisition of a tumor-infiltrating T_reg_ cell phenotype (24).

In an effort to better understand the role of MEOX1 in T_reg_ cells, we analyzed the MEOX1 locus and demonstrated that it is epigenetically more accessible in T_reg_ cells than in all other CD4^+^ T cell subsets and that it contains a FOXP3 binding site. Furthermore, we validated these data in human T_reg_ cells at both the mRNA and protein level, and by siRNA knockdown experiments established that transcriptional control through MEOX1 is downstream of FOXP3. Using transcription factor binding prediction, we identified target genes of MEOX1 which we experimentally verified to be MEOX1 target genes at the mRNA level. Furthermore, we could link expression of MEOX1 to T_reg_ cell suppressive function. Thus, by applying a systems immunology approach we discovered a new transcription factor in human T_reg_ cells operating downstream of FOXP3 important for human T_reg_ cell activity.

## 2 Materials and methods

### Key resources table

**Table:** 

REAGENT or RESOURCE	SOURCE	IDENTIFIER
Antibodies		
Unconjugated anti-human CD3 Antibody, Clone OKT3	Ortho Biotech	Cat# OKT3, RRID : AB_2619696
Unconjugated anti-human CD28, Clone 9.3	Kind gift from J.L. Riley	RRID : AB_2687729
Unconjugated anti-human MHC I Antibody, Clone W6/32	Thermo Fisher Scientific	Cat# MA1-22572, RRID : AB_560084
PerCP/Cy5.5 anti-human CD127 (IL-7R) Antibody, Clone A019D5	Biolegend	Cat# 351321, RRID : AB_10900253
APC anti-human CD127 (IL-7R) Antibody, Clone A019D5	Biolegend	Cat# 351342, RRID : AB_2564137
Brilliant Violet 510 anti-human CD25 Antibody, Clone BC96	Biolegend	Cat# 302640, RRID : AB_2629672
PE anti-human CD25 Antibody, Clone BC96	Biolegend	Cat# 302606, RRID : AB_314276
Brilliant Violet 421 anti-human CD3 Antibody, Clone UCHT1	Biolegend	Cat# 300434, RRID : AB_10962690
FITC anti-human CD3 Antibody, Clone UCHT1	Biolegend	Cat# 300452, RRID : AB_2564148
PE/Cyanine7 anti-human CD4 Antibody, Clone RPA-T4	Biolegend	Cat# 300512, RRID : AB_314080
PE/Dazzle 594 anti-human CD4 Antibody, Clone RPA-T4	Biolegend	Cat# 300548, RRID : AB_2563566
PE anti-human FOXP3 Antibody, Clone PCH101	eBioscience	Cat# 12-4776-42, RRID : AB_1518782
PE anti-human FOXP3 Antibody, Clone 206D	Biolegend	Cat# 320108, RRID : AB_ 492986
FITC anti-human CD45RA Antibody, Clone HI100	BD Biosciences	Cat# 555488, RRID : AB_395879
Unconjugated anti-mouse/human Actin Antibody, clone C4 antibody	Millipore	Cat# MAB1501, RRID : AB_2223041
Unconjugated anti-human MEOX1 Antibody, rabbit polyclonal antibody	Abcam	Cat# ab23279, RRID : AB_447360
IRDye 800CW goat anti-mouse IgG Antibody, polyclonal goat anti-mouse IgG	LI-COR Biosciences	Cat# 926-32210, RRID : AB_621842
IRDye 680RD goat anti-rabbit IgG antibody, polyclonal goat anti-rabbit IgG	LI-COR Biosciences	Cat# 925-68071, RRID : AB_2721181
Alexa Fluor 647 goat anti-rabbit IgG (H+L), polyclonal recombinant goat anti-rabbit IgG	Thermo Fisher Scientific	Cat# A27040, RRID : AB_2536101
Biological Samples		
Buffy-coat samples	Universityhospital, Bonn	Reg. No. 288/13
Chemicals, Peptides, and Recombinant Proteins		
Superscript II Reverse Transcriptase	Thermo Fisher Scientic	Cat# 18064014
KAPA HiFi HotStart ReadyMix	Kapa Biosystems	Cat# KR0370
High Sensitivity D5000 ScreenTape	Agilent Technologies	Cat# 5067-5592
High Sensitivity D5000 Reagents & Ladder	Agilent Technologies	Cat# 5067-5593
Recombinant human TGF-β1 (HEK293 derived)	PeproTech	Cat# 100-21
IL-2 (Proleukin)	Chiron/Novartis	PZN# 2238131
cOmplete Protease Inhibitor Cocktail	Roche	Cat# 04693116001
PhosSTOP™	Roche	Cat# PHOSS-RO
SuperScript III One-Step RT-PCR System with Platinum Taq High Fidelity DNA Polymerase	Thermo Fisher Scientific	Cat# 12574030
MyTaq HS DNA Polymerase	Boline	Cat# BIO-21111
CFSE	eBioscience	Cat# 65-0850-84
Cell Proliferation Dye eFluor 670	eBioscience	Cat# 65-0840-85
Critical Commercial Assays		
Foxp3/Transcription Factor Staining Buffer Set	eBioscience	Cat# 00-5523-00
CD4 M-pluriBead anti-human M-kit	Pluriselect	Cat# 19-00400-20 and 70-50010-21
RosetteSep Human CD4+ T Cell Enrichment Cocktail	Stem Cell	Cat# 15022
CD25 MicroBeads II, human	Miltenyi Biotec	Cat# 130-092-983
CD45RA MicroBeads, human	Miltenyi Biotec	Cat# 130-045-901
Universal ProbeLibrary Set, Human	Roche	Cat# 4683633001
Transcriptor First Strand cDNA synthesis kit	Roche	Cat# 4897030001
Maxima SYBR Green Master	Thermo Fisher Scientific	Cat# K0221
LightCycler 480 Probes Master	Roche	Cat# 4887301001
miRNeasy Mini Kit	Qiagen	Cat# 217004
QIAquick Gel Extraction Kit	Qiagen	Cat# 28704
QIAquick PCR Purification Kit	Qiagen	Cat# 28104
Human T Cell Nucleofector Kit	Lonza	Cat# VPA-1002
LIVE/DEAD Fixable Near-IR Dead Cell Stain Kit	Thermo Fisher Scientific	Cat# L10119
Deposited Data		
Tiling array Data	(25)	GSE20995
Microarray Data	(10, 26)	GSE15390 and GSE17241
MeDIP-seq Data	http://trace.ddbj.nig.ac.jp	DRP000902
Histone Modification Dataset	GEO NIH Roadmap Epigenomics	http://www.roadmapepigenomics.org
FOXP3 ChIP-seq data	(27)	SRA : SRP006674
Human Immune Cell Dataset	www.nextbio.com	n/a
Microarray dataset	(11)	GSE11292
scRNA-seq data	(28)	GSE99254
Experimental Models: Cell Lines		
HEK293T	ATCC	Cat# CRL-3216, RRID : CVCL_0063
Oligonucleotides		
For Oligonucleotide Sequences, see **Supplemental Table S7**	This paper	n/a
Software and Algorithms		
FlowJo	BD Biosciences	RRID : SCR_008520
LightCycler Software	Roche	RRID : SCR_012155
GenomeStudio	Illumina	RRID : SCR_010973
BioLayout Express 3D	http://www.biolayout.org	RRID : SCR_007179
Partek Genomics Suite (PGS)	Partek	RRID : SCR_011860
R 3.4.1	http://www.r-project.org/	RRID : SCR_001905
genomics Workbench 2.6.0	http://www.geworkbench.org	RRID : SCR_013599
Cytoscape	http://cytoscape.org	RRID : SCR_003032
LegumeGRN	http://legumegrn.noble.org	n.a.
ToppGene Suite	http://toppgene.cchmc.org/	RRID : SCR_005726
Integrative Genomics Viewer	http://www.broadinstitute.org/igv/	RRID : SCR_011793
BD FACSDiva Software	http://www.bdbiosciences.com/instruments/software/facsdiva/index.jsp	RRID : SCR_001456
Graph Pad Prism	GraphPad Software	RRID : SCR_002798
Fiji ImageJ	http://fiji.sc	RRID : SCR_002285

### 2.1 Experimental model and subject details

#### 2.1.1 Human subjects

Human T cells were purified from Buffy coats of healthy human donors obtained in compliance with institutional review board protocols (Ethics committee, University of Bonn, Reg. No. 288/13) after written consent. Due to privacy regulations, gender and age of these donors could not be ascertained.

#### 2.1.2 Cell lines

Human embryonic kidney (HEK) 293T (ATCC CRL-11268; female) cells were maintained in DMEM containing 10% heat-inactivated fetal calf serum. Cells were cultivated at 37°C, 5%CO2.

### 2.2 Method details

#### 2.2.1 Dataset compilation and primarydata handling

All microarray gene expression data (GSE15390 and GSE17241) were downloaded from the GEO database and compiled using GenomeStudio (Illumina). A total of 217 samples were imported into Partek Genomics Suite 5.0 (PGS) for further analysis including quantile normalization. Batch effects caused by separate array experiments were removed from normalized log2-transformed data. Background signal was calculated within R based on the coefficient of variation (the computed background for the entire dataset was 7.183). Genes were defined as expressed and kept for further analyses if their mean expression values were higher than background in at least one of the 217 samples. Afterwards, multi-probes were filtered to retain only a single probe with the highest mean expression as representative for the corresponding gene. To this end, principal component analysis (PCA) was performed and validated using networks based on Pearson’s correlations which were visualized in BioLayout Express3D. Only samples, which clearly deviated from other samples in both methods, were assumed to be outliers and hence removed from the dataset. Finally, 217 samples containing 14,632 expressed genes (also referred to as present genes) were kept for further analyses in R 3.4.1. Validation of MEOX1 expression in human T_reg_ cells was performed by reanalyzing a publicly available dataset downloaded from GEO (GSE11292) (11).

#### 2.2.2 Principal component analysis and t-distributed stochastic neighbor embedding

PCA was applied on all present genes using the function prcomp of the R package stats by leaving the default setting unaltered. Moreover, nonlinear dimensionality reduction was performed to identify similarities between the CD4^+^ T cell samples by utilizing t-distributed stochastic neighbor embedding (t-SNE) (29). Therefore, the R package Rtsne was applied to all present genes by leaving the standard parameters unaltered despite of theta which was set to 0.0.

#### 2.2.3 Correlation coefficient matrices combined with hierarchical clustering

The computation of the Pearson’s correlation coefficients (PCC) was done in a pairwise fashion between all CD4^+^ T cell conditions using PGS, which resulted in correlation coefficient matrices (CCMs). PCCs were computed using Pearson’s (Linear) correlation based on all present genes. The hierarchical clustering is based on Euclidean distance of the cells and was plotted as the standardized correlation coefficients (mean of zero and standard deviation of one) for the CD4^+^ T cell conditions. This resulted in 11 larger clusters representing all 48 conditions.

#### 2.2.4 ANOVA calculation for differential gene identification

Data were analyzed in PGS by 2-way ANOVA for more than two CD4^+^ T cell conditions and student’s t-test for two conditions only. DE genes were defined by the 2-way ANOVA model (|FC| > 2, FDR adjusted p-value < 0.05) (30). The identified DE-genes were visualized in an UpSet plot using the R package ‘UpSetR’ (31).

#### 2.2.5 K-means clustering combined with hierarchical clustering

In accordance to k-means clustering performed by Smeekens et al. (32) we used as input to the algorithm the most variable genes out of the 2-way ANOVA (p < 0.05; 9,925 genes) and calculated the fold-change (FC) between any sample and freshly isolated naïve CD4^+^ T cells (also referred to as cluster 11 or “T_conv_ cell resting”). To determine the optimal number of k clusters, the Davies-Bouldin index of absolute expression values was determined using PGS which resulted in 25 clusters. Similar to CCMs, the hierarchical clustering was calculated followed by ranking of the rows according to k-means cluster-affiliation and plotting of the standardized fold-changes (mean of zero and standard deviation of one) for the CD4^+^ T cell conditions. This resulted in 10 larger clusters representing all 48 conditions.

#### 2.2.6 Self-organizing map clustering

Using the SOM-clustering implementation of PGS, the CCM-defined clusters were compared based on topological patterns and enabled the investigation of cluster-specific genes. First, the mean expression of the most variable genes based on a 2-way ANOVA was calculated for each cluster separately and standardized to a mean of zero and standard deviation of one. Next, 20,000 training iterations were used to cluster similar genes close to each other on the map. In our settings, the 9,925 genes were divided into 10 x 10 SOM-clusters (approximately 90 genes in each SOM-cluster), and the mean expression values of each SOM-cluster genes were used to calculate an eigenvalue, which represented the general expression value of this SOM-cluster. The resulting data were then visualized as a heatmap representing increased values in red, decreased values in blue and intermediate values in green.

#### 2.2.7 Weighted gene co-expression network analysis

We utilized the WGCNA R package (33) to identify co-expressed genes associated with the 11 CCM-defined clusters. As input to this algorithm served the union of all DE-genes (|FC| > 2, FDR adjusted p-value < 0.05) between a certain cluster and the ‘T_conv_ cell resting’-cluster. The standard parameter of WGCNA was altered to a power of 15 and a minModuleSize of 10 resulting in 32 modules. For each module, the eigengene corresponding to the first principal component was calculated and subsequently correlated to the respective clusters. The correlation values were visualized in a heatmap.

#### 2.2.8 Gene set enrichment analysis

To validate WGCNA, GSEA on the 32 modules in 10,000 permutations using PGS was performed (34). For each comparison (samples within a CCM-defined cluster versus all other samples of the dataset), normalized enrichment score (NES), allowing comparisons of overrepresentation between different gene sets, together with p-value of GSEA were plotted by Volcano plots. The two WGCNA modules, which exhibited both the highest eigengene-to-cluster correlation and a significant p-value (< 0.05), based on GSEA were selected. Genes within these modules were visualized in another Volcano plot by plotting expression ratios (reference: ‘T_conv_ cell resting’) versus p-values obtained by t-test statistics.

#### 2.2.9 Prediction of potential FOXP3 targets

The Cytoscape plug-in iRegulon (35) was used to investigate the potential upstream transcription factors (TFs) controlling the expression of genes found within the two WGCNA modules with the highest correlation to ‘T_reg_ cell CD3/IL2’. Therefore, TF prediction was performed in a genomic region 500 bp upstream of the TSS. Subsequently, all genes which exhibited binding motifs for FOXP3 were visualized in a circular layout in Cytoscape.

#### 2.2.10 Prediction of potential MEOX1 targets

To identify potential MEOX1 target genes, we utilized the database provided by the R package tftargets (https://github.com/slowkow/tftargets ) and queried genes that carry a binding site for MEOX1 in their promoter regions. The results were then filtered for genes found within the two WGCNA modules with the highest correlation to ‘T_reg_ cell CD3/IL2’ and that exhibited an expression fold change > |1.0|.

#### 2.2.11 Algorithm for the Reconstruction of Accurate Cellular Networks (ARACNe)

The expression data was loaded into an integrated genomic analysis platform genomics Workbench 2.6.0 (36) to implement the ARACNe algorithm for network analysis (37). All present genes were taken into calculation of mutual information (MI) with p-values less than 0.05 without Bonferroni correction. The threshold of data processing inequality (DPI) theorem from information theory was set to 0.01. The resulting network consisted of 14,494 nodes with 179,876 edges

#### 2.2.12 Tool for inferring network of genes (TINGe)

Similar to ARACNe, all present genes were used as input to TINGe (38). However, we used neither a p-value cutoff nor a DPI-value. The resulting network consisted of 14,494 nodes with 235,933 edges.

#### 2.2.13 Context likelihood of relatedness (CLR)

We took advantage of the web server LegumeGRN that provides a CLR-implementation (39). We used the CLR-method (“plos”) which was used in the original publication (40) and left the default setting unaltered with the exception that the number of edges in the output file was limited to 250,000. The resulting network consisted of 12,641 nodes.

#### 2.2.14 Gene network inference with ensemble of trees (GENIE3)

In the present study, we applied the R package genie3 (41) for the prediction of the regulatory network of all present genes by setting the number of trees to 1000 and limit report.max to 250,000. The resulting network consisted of 12,302 nodes with 227,449 edges.

#### 2.2.15 Basic correlation

Pearson’s correlation was employed to compute the relationships between all gene pairs within the gene expression data. As input to this calculation, we used genes which were found in the output-networks of all of the abovementioned algorithms (10,721 genes). Pearson’s correlation was calculated using BioLayout Express3D. Setting the correlation cutoff to 0.7 resulted in a network consisted of 7,216 nodes with 700,136 edges.

#### 2.2.16 Consensus network

All gene-gene interactions derived from each individual network prediction were ranked by its ranking function. By computing average rank for each gene pair using the GP-DREAM module AverageRank (42), 10,000 top ranking interactions was obtained. The resulting consensus network consisted of 3,845 nodes (R^2^ = 0.938) and was visualized in a force-directed layout in Cytoscape.

#### 2.2.17 Candidate gene prioritization

To link identified hub genes with transcriptional regulation of T cells, we supplemented the consensus network obtained by AverageRank with prior knowledge by applying the following strategy. The top 20% highly connected hub genes were prioritized by association with transcriptional regulation of T cells using the well-known T cell biology regulators TCF7, SATB1, EZH2 and GATA3 as test genes for the prioritization tools ToppGene (43) and Endeavour (44). As training parameters of ToppGene we used following features: “GO: Molecular Function”, “GO: Biological Process”, “GO: Cellular Component”, “Pathway”, “Pubmed”, “Coexpression” and “Coexpression Atlas”. For Endeavour following features were used: “EnsemblEst”, “GeneOntology”, “Kegg”, “Swissprot”, “Expression – SonEtAl” and “Expression – SuEtAl”. The results of the two approaches were combined by the Borda ranking method.

#### 2.2.18 Identification of target genes of prioritized TFs using iRegulon

The 20% hub genes were used as input to iRegulon and potential upstream TFs were predicted in a genomic region of 500 bp of their respective TSS. Subsequently, the predicted TFs CREB1, E2F3, AHR, STAT1, NFAT5 and NFATC3 and their putative target genes were visualized in a network using the circular layout tool of Cytoscape. Moreover, the prioritized TFs and their target genes were used to perform GOEA.

#### 2.2.19 Clustering of the consensus network using Markov clustering algorithm (MCL)

To identify sub-structures within the consensus network, we applied MCL which is implemented in the Cytoscape plug-in clusterMaker (45). In the present study we applied MCL using the default settings.

#### 2.2.20 Single-cell RNA-seq analysis

We downloaded a recently published T cell single-cell RNA-seq dataset from the GEO database (GSE99254) that comprises CD4^+^ T cell- and CD8^+^ T cell-populations from non-small-cell lung cancer patients (28). The published t-SNE map of the single-cell study was reconstructed by utilizing the normalized and centered data provided on the GEO database and following the recommended pipeline of the authors. Briefly, we took advantage of the sscClust analysis pipeline (https://github.com/Japrin/sscClust) with which we determined the 1,500 most variable genes using the “sd” parameter followed by the calculation of the top 30 principal components, which were then used as input into the t-SNE construction. The cluster-annotation was extracted from published analysis results provided on the webpage http://lung.cancer-pku.cn. Identification of MEOX1 co-expressed genes in the single-cell RNA-seq dataset was performed using the TPM normalized expression data as input and calculating the Pearson’s correlation of MEOX1 with all other genes.

#### 2.2.21 Analysis of histone modifications of the genomic MEOX1 locus

ChIP-seq data, which were provided by the NIH Roadmap Epigenomics Mapping Consortium, (46), was downloaded as WIG (wiggle) file and visualized using the integrative genomics viewer (IGV). For the analysis we used the available information about histone modifications for CD4^+^CD25^-^CD45RA^+^ T cells and CD4^+^CD25^+^CD127^-^ T cells (both cell-types obtained from donor 63).

#### 2.2.22 Isolation of human T_reg_ and T_conv_ cells from buffy coats

Human T cells were purified from Buffy coats of healthy human donors. CD4^+^ T cells were isolated by positive selection using pluribeads (Pluriselect) according to manufacturer’s instructions. To isolate T_reg_ and T_conv_ cells, cells were either isolated using CD25 MACS beads (Miltenyi Biotech) or sorted on a BD Aria III cell sorter (BD Biosciences) after staining with antibodies against CD3, CD4, CD25 and CD127. Gating strategy is shown in **Figures S2A, B**. As MEOX1 is an intracellular protein, during sorting for living cells it was not possible to stain for the expression of MEOX1. In addition, dead cells were excluded using the LIVE/DEAD fixable near-IR dead cell stain kit (Thermo Fisher Scientific).

#### 2.2.23 Transfection of MEOX1

MEOX1 vector was transfected into HEK cells using the Turbofect transfection reagent (Thermo Fisher) according to manufacturer’s instructions. Transfection efficacy was tested by flow cytometry and only cells with a transfection efficacy of ≥85% were used for subsequent experiments.

#### 2.2.24 Flow cytometric analysis

Antibodies for flow cytometric analyses were purchased from Biolegend or Thermo Fisher Scientific. Extracellular staining was performed at 4°C in the dark for 30 minutes. Intracellular staining of FOXP3 was performed using the Foxp3 Staining Buffer kit (Thermo Fisher Scientific) according to manufacturer’s instructions using the PCH101 clone for unstimulated T_reg_ cells, while staining of FOXP3 in stimulated T_reg_ cells was performed with the 206D clone. MEOX1 staining for flow cytometry was performed by first staining for MEOX1 for 1 hour at 4°C followed by staining with a fluorochrome conjugated anti-rabbit secondary antibody for 30 minutes at 4°C in the dark. Samples were acquired on a BD LSR II or Symphony A5 flow cytometer (BD Biosciences). Data were analyzed using FlowJo.

#### 2.2.25 IL-2 stimulation

For IL-2 stimulation, MACS-isolated T_reg_ cells were cultured in 96 well plates at a concentration of 1x10^5^ cells/well in X-Vivo 15 medium in the presence of 0, 10, 100 or 1000 U/ml IL-2 for 0, 12, 24, 48 or 72 hours. Cells were subsequently harvested in Trizol (Thermo Fisher Scientific) prior to RNA isolation. RNA from higher cell numbers was isolated by isopropanol precipitation as previously described (47). For flow cytometry staining of MEOX1, PBMCs were isolated and seeded in 6 well plates at a concentration of 4x10^6^ cells/well in 3 ml RPMI stimulated with 100 U/ml IL-2 overnight.

#### 2.2.26 RNA isolation, cDNA synthesis and qRT-PCR

Cells were resuspended in Trizol (Thermo Fisher Scientific) and RNA was isolated according to the manufacturer’s recommendations. If fewer than 50.000 cells were used for RNA isolation, the miRNeasy Mini Kit (Qiagen) was used instead according to the manufacturer’s instructions. cDNA was generated using the Transcriptor First Strand cDNA synthesis kit (Roche Diagnostics) according to manufacturer’s specifications. qRT-PCR was performed using LightCycler Taqman master kit and the Universal Probe Library assay (Roche Diagnostics). Primer Sequences are listed in **Table S7**.

#### 2.2.27 siRNA-mediated knockdown

siRNAs were purchased from Biomers and used for transfection of MACS-isolated T_reg_ cells. Transfection was carried out with the human T cell nucleofector kit (Lonza) as per manufacturer’s specifications as previously described (10). Sequences of siRNAs can be found in **Table S7**.

#### 2.2.28 DNA methylation analysis

Genomic DNA was isolated from sorted and stimulated T_reg_ and T_conv_ cells using the DNeasy Blood & Tissue Kit (Qiagen) and concentrated using the DNA Clean & Concentrator Kit (Zymo Research), both following the manufacturer’s instructions. The sample DNA was converted by bisulfite using the EZ DNA Methylation-Lightning Kit (Zymo Research) according to the manufacturers protocol and subjected to pyrosequencing as described previously (48). Amplification and sequencing of the regions A (chromosome position17: 43662392-43662439) and B (chromosome position 17: 43661827-43661888) in the MEOX1 gene locus was performed with the amplification/sequencing primers listed in **Table S7**. The indicated chromosome positions refer to genome assembly GRCh38.p13.

#### 2.2.29 Immunoblotting

Immunoblotting was performed as previously described (12). Briefly, cells were lysed in RIPA buffer (10 mM Tris-Cl (pH 8.0), 1 mM EDTA, 0.5 mM EGTA, 1% Triton X-100, 0.1% sodium deoxycholate, 0.1% SDS, 140 mM NaCl) Lysates were denatured and run on a 10% SDS-PAGE gels and blotted onto nitrocellulose membranes. Primary AB incubation was performed in 5% milk in PBST or 2.5% BSA in PBST, according to AB manufacturer recommendations for at least 12 hours at 4°C. Blots were then incubated with fluorochrome coupled secondary AB. Following the secondary antibody incubation, protein signals were detected on the LICOR Odyssey Imaging System. The following antibodies were used: MEOX1 (abcam) ab23279, Actin (Sigma-Aldrich) MAB1501.

### 2.3 Quantification and statistical analysis

All statistical analysis except analysis of gene expression data were performed with Graph Prism software version 8.0 (GraphPad Software). Unless otherwise specified, data were plotted as mean ± SEM. To determine significant differences the two-tailed student’s *t* test was performed when comparing normally distributed data of two groups or *post-hoc* Bonferroni when comparing multiple groups. P values less than 0.05 were considered significant (n.s. indicates not significant, * = p < 0.05).

### 2.4 Data and software availability

GEO: microarray data, GSE15390 and GSE17241; tiling array data, GSE20995; public datasets: MeDIP-seq data, DRP000902 (http://trace.ddbj.nig.ac.jp); Histone modification dataset, GEO NIH Roadmap Epigenomics (http://www.roadmapepigenomics.org); FOXP3 ChIP-seq data: SRA: SRP006674; Human immune cell dataset, www.nextbio.com; Validation of MEOX1 expression in human T_reg_ cells: GSE11292. Single-cell RNA-seq: GSE99254.

## 3 Results

### 3.1 Transcriptome analysis of CD4^+^ T_conv_ and T_reg_ cells reveals activation-dependent clustering of cells

To better understand how activation influences gene expression in T_conv_ and T_reg_ cells and to identify key regulatory events responsible for T cell differentiation, we analyzed the transcriptome of human resting or activated T_conv_ and natural T_reg_ cells together with T_H_1 and T_H_2 differentiated cells (**Table S1**). To do this, we combined data from our previous work (10) with an analysis focusing on T_H_1- and T_H_2-differentiation of CD4^+^ T cells (26) resulting in a dataset comprising 48 different conditions.

**Figure 1:**
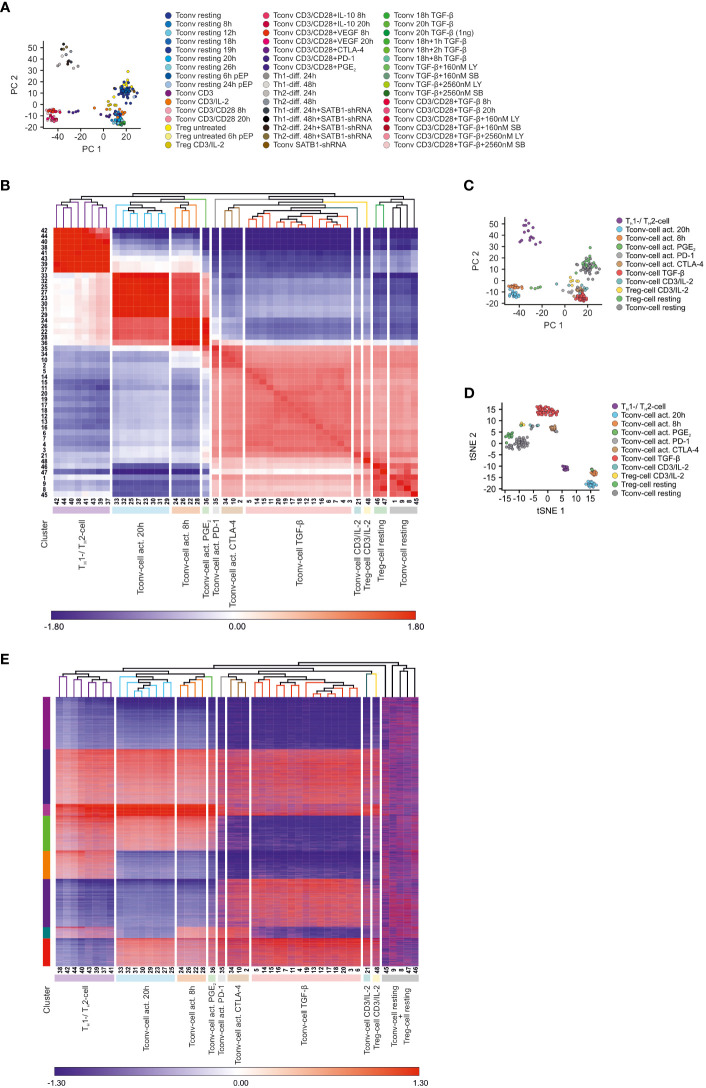
Data dimensionality reduction reveals transcriptional relationships between different CD4^+^ T cell samples **(A)** Visualization of the dataset by depicting the first (PC1) and the second principal components (PC2) of the computed PCA annotated with all 48 conditions. **(B)** Z-score normalized matrix of hierarchically clustered Pearson’s correlation coefficients between CD4^+^ T cell conditions. Conditions were annotated according to **Table S1**. Clusters of transcriptional similar CD4^+^ T cell conditions were annotated according to the predominant stimulation among the conditions. **(C)** PCA annotated according to the predominant stimulation among the conditions. **(D)** Visualization of the dataset using t-SNE. **(E)** Visualization of k-means clusters combined with hierarchical clustering. As input to k-means clustering served expression differences (fold changes with resting T_conv_ cells as reference), which were calculated for the most variable genes within the dataset. Z-score normalized fold changes are indicated by the coloring (blue to red). Conditions were annotated according to **Table S1**.

To gain insight into the general sample-to-sample relationships, we first visualized the transcriptional variance of the complete dataset using principal component analysis (PCA, **Figure 1A**). This analysis revealed a bipolar structure in which cells stimulated by CD3/CD28 antibodies (activated T cells) clustered away from all other T cells, indicating that the activation stimulus is causing the highest variance within the dataset. Moreover, this analysis further indicated that the global transcriptional changes induced by CD3/CD28 stimulation were so prominent, that co-incubation with putative inhibitory molecules like IL-10, TGF-β, and VEGF were not able to revert or modulate this response. The only exceptions to this observation were activated CD4^+^ T cells which were co-incubated with blocking antibodies against immune checkpoint molecules, such as PD-1 or CTLA-4, as these cells exhibited a close transcriptional relationship to resting CD4^+^ T cells in the PCA. Interestingly, when plotting first and second principal components, CD3/CD28-activated CD4^+^ T cells were separated into two populations. Activated CD4^+^ T cells stimulated by plate-bound CD3 and soluble CD28 in combination with T_H_1- and T_H_2-polarizing cytokines formed a distinct population (26), while CD4^+^ T cells stimulated with CD3/CD28-coated beads were clearly separated from this population (10). Within the population of CD4^+^ T cells not activated by CD3/CD28, there was also a separation detectable between cells cultured *in vitro* for more than 12 hours and cells harvested directly after or within 12 hours after isolation. Unexpectedly, T_reg_ cells were grouped together with either the unstimulated or short-term cultivated cells, further supporting that the major difference between samples was driven by activation. In summary, using multidimensional scaling we were able to identify several distinct sample clusters within the dataset, with TCR/CD28 activation being the main driver of biological variance while lineage defining aspects, e.g. differences between T_conv_ and T_reg_ cells only contributing a small portion to the overall variance.

### 3.2 Reduction of data complexity using correlation coefficient matrices identifies subclusters of transcriptionally related CD4^+^ T cells

Since the PCA indicated that the samples within the dataset exhibited different degrees of transcriptional relationships and thus formed distinct clusters, we reasoned that we could reduce the complexity of the dataset by grouping transcriptionally related samples together and performing further analyses using these clusters, instead of analyzing each sample separately. To this end, we computed Pearson’s correlation coefficients between all conditions and visualized the results in a correlation coefficient matrix (CCM) combined with hierarchical clustering (HC) (**Figure 1B**). This analysis revealed that the samples clustered in a bipolar fashion, grouping activated separately from non-activated CD4^+^ T cells, supporting the observation that CD3/CD28 activation caused the biggest variance within the dataset. However, the agglomerative nature of HC enabled us to refine the bipolar structure of the dataset by identifying several sub-clusters. More specifically, we found that the complete dataset was characterized by at least 11 clusters. Next, cluster names were selected representing the majority of T cell conditions within the clusters (**Table S2**) and mapped to the PCA (**Figure 1C**). For example, the cluster on the right hand side was mainly composed of untreated CD4^+^ T cells and hence was termed ‘T_conv_ cell resting’. As this cluster represented the cell state with the lowest degree of activation within the dataset, we were surprised that these cells were most closely related to T_reg_ cells.

To substantiate the results obtained by the hierarchical clustering of the CCM, we visualized the data structure by utilizing t-Distributed Stochastic Neighbor Embedding (tSNE) as a non-linear data reduction approach and colored the samples according to their assignments in the respective CCM clusters (**Figure 1D**). We observed that the tSNE clusters nicely overlapped with the CCM clusters. Moreover, we also corroborated the validity of the CCM clusters by applying K-means clustering (**Figure 1E**). Taken together, the samples within the dataset were successfully grouped into biologically informative clusters based on their transcriptional profile.

### 3.3 Identification of common denominators of CD4^+^ T cell functionality using reverse network engineering

**Figure 2:**
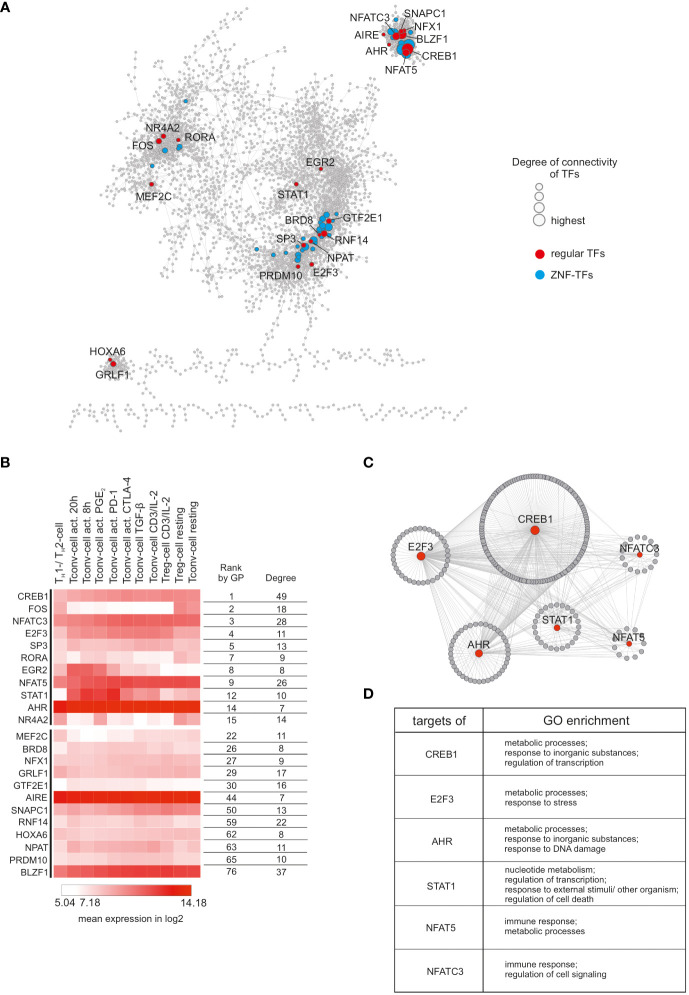
Reverse network engineering to infer common CD4^+^ T cell genes. **(A)** Visualization of the consensus network obtained by the combination of five different RNE-methods. TFs found among the top 20% hub genes were highlighted (common/regular TFs in red; ZNF-TFs in blue). Node size reflects degree of connectivity. **(B)** Top 23 highest interconnected common TFs were ranked according to Gene Prioritization (GP) among the top 20% hubs. Mean expression (log2) from each cluster is displayed as a heatmap. Degree refers to degree of connectivity. **(C)** Subnetworks of the six highest expressed TFs from the top 11 GP-ranked hubs. Direct targets (predicted by iRegulon) surround corresponding TFs. Node size reflects degree of connectivity.

To identify the key genes for general CD4^+^ T cell functionality, we took advantage of reverse network engineering (RNE). To obtain a robust interaction network, we generated a consensus network by combining the following network inference methods: ARACNe (Algorithm for the Reconstruction of Accurate Cellular Networks), CLR (Context Likelihood of Relatedness), GENIE3 (Gene Network Inference with Ensemble of Trees), Pearson’s correlation networks, and TINGe (Tool for Inferring Network of Genes). Next, we combined the generated networks and used the 10,000 top ranking interactions of the resulting consensus network for further analysis (remaining gene/node number: 3845). As expected, given the scale-free nature of the network, a small percentage of genes accounts for most of the connections and thus are referred to as hub genes. We defined as major hub genes the largest 20% of hubs in the network which collectively participate in 7,948 interactions (**Figure 2A**). As we were interested in transcriptional key regulators of CD4^+^ T cell functionality, we used the TFCat database (49) for filtering the consensus network. Among the top 20% hub genes, we identified 23 common TFs and additional 43 zinc finger (ZNF) TFs (e.g. ZNF454, ZNF549, and ZNF136). Interestingly, we found that the TFs tended to form clusters within the network, suggesting a complex regulatory TF network in which TFs were strongly co-regulated (**Figure 2A**). To further reduce the list of important TFs for CD4^+^ T cell functionality, we applied two gene prioritization tools, ToppGene and Endeavour, on the top 20% hub-genes. As internal validation gene set, we decided to use the transcriptional regulators TCF7, SATB1, GATA3 and EZH2, which were already described to be important during T cell development, chromatin-organization, T cell differentiation, and T cell homeostasis, respectively (10, 50–53). Of note, 11 transcription factors (TFs) were found among the top 15 highest prioritized genes (**Figure 2B**). Reasoning that of those, the most highly expressed TFs within the dataset are the most relevant for CD4^+^ T cell functionality, we identified six central TFs (CREB1, E2F3, AHR, STAT1, NFAT5 and NFATC3). For all of them, important roles in T cell functionality have already been described (54–59). To investigate genes which were presumed to be regulated by these TFs, we used the top 20% hub genes as input to iRegulon. As expected, for all six TFs, a relatively high prediction score (normalized enrichment scores (NES) > 2.5) was computed which indicated that a multitude of the top 20% hub genes contained binding-motifs for these TFs. Next, we generated a network of the six TFs and their putative targets (**Figure 2C**). Remarkably, the network was highly interconnected and showed binding of the TFs between each other, supporting the idea of a sophisticated regulatory network built by TFs which control the central CD4^+^ T cell functionality. To link the genes within the network to biological information, we performed Gene Ontology Enrichment Analysis (GOEA) for each TF together with its direct neighbors (**Figure 2D**, **Table S3**). Most of the enriched GO-terms were related to regulation of metabolism and transcription, clearly supporting that metabolic and transcriptional adaptation are key events upon T cell activation (60, 61). In addition, NFAT5 and NFATC3 were found to regulate genes associated with immune response which is in line with previous observations that NFAT family members are critically involved in the induction of a T cell mediated adaptive immune response (54, 57, 62). Taken together, RNE analysis identified six putative central transcriptional regulators of CD4^+^ T cell functionality.

### 3.4 Identification of genes specifically associated with distinct CCM clusters

**Figure 3:**
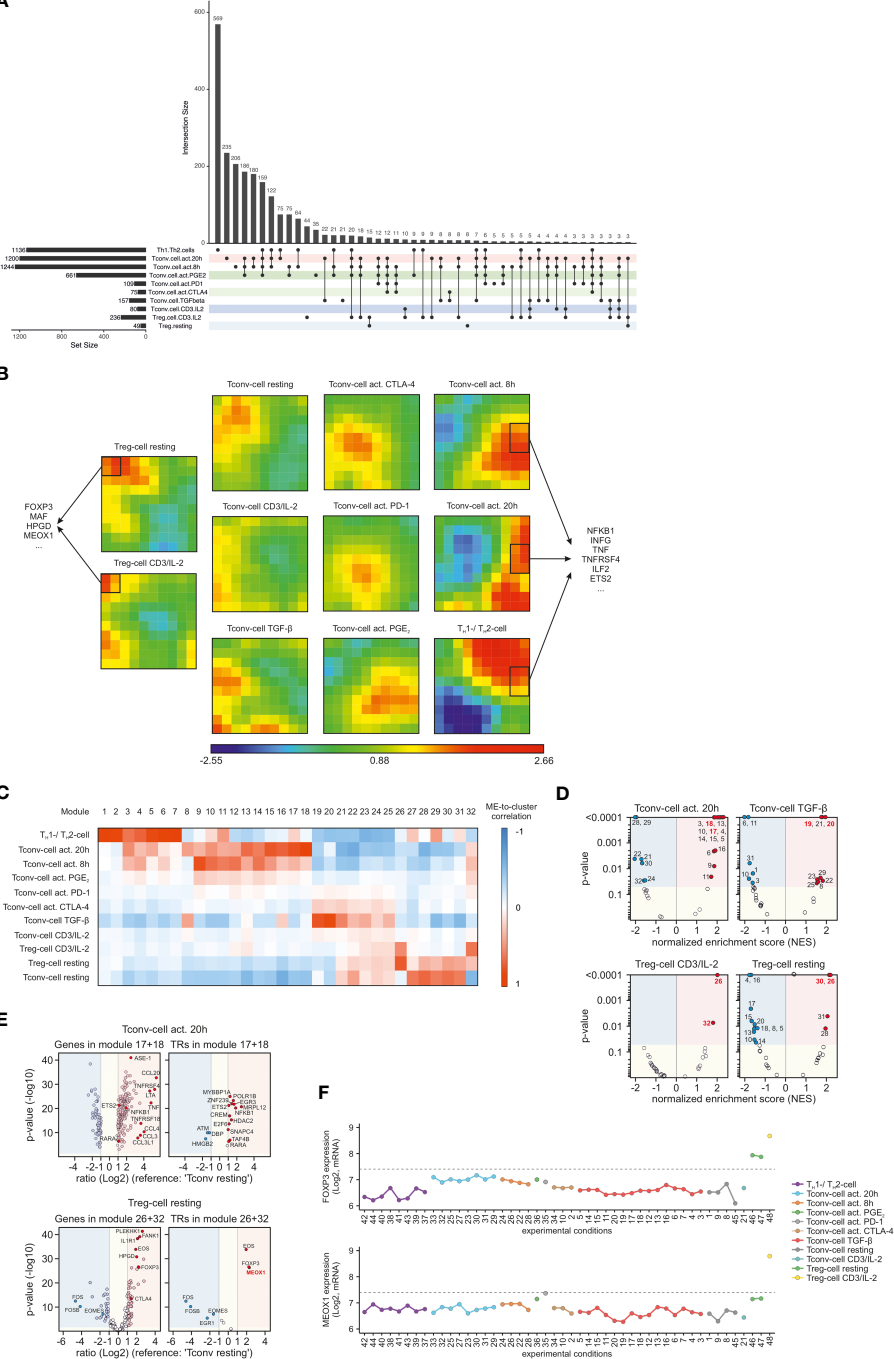
Identification of genes associated with the identified CCM clusters **(A)** UpSet plot of calculated DE genes across the CCM clusters. DE genes found in the same clusters are binned and the size of the bins is represented as a bar chart. At the bottom, dots indicate which CCM clusters contained and shared these DE genes. Only bins with >2 DEgenes are depicted. **(B)** SOM-clustering using the most variable genes within the dataset as input. Correlation of SOM-cluster genes to CCM-defined clusters are indicated by z-scaled color coding; blue indicates low correlation and red indicates high correlation. SOM clusters specific for either T_reg_ cells or activated CD4^+^ T cells are marked with a black frame; exemplary genes within these clusters are displayed. **(C)** WGCNA heatmap showing the correlation of the module eigengene (first principal component; ME) to the traits (CCM clusters). Blue means negative correlation and red means positive correlation. **(D)** Volcano plots of normalized enrichment scores (NES) and enrichment p-values based on GSEA using WGCNA modules defined in **(C)**. Shown are data for the clusters ‘T_conv_ cell act. 20h’, ‘T_conv_ cell TGF-β’, ‘T_reg_ cell CD3/IL-2’ and ‘T_reg_ cell resting’. Red circles show significantly enriched gene sets; blue circles show significantly depleted gene sets. Gene sets which exhibited the highest correlation to a certain cluster in WGCNA are indicated by red font. **(E)** Volcano plots genes within the two WGCNA modules with the highest correlation to the CCM cluster ‘T_conv_ cell act. 20h’ and ‘T_reg_ cell CD3/IL-2’. Depicted are the logarithmic gene ratios (‘T_conv_ cell act. 20h’ or ‘T_reg_ cell CD3/IL-2’ versus ‘T_conv_ cell resting’) and logarithmic p-values obtained by t-test. Red circles show upregulated genes (FC >2; p-value <0.05); blue circles show downregulated genes (FC <-2; p-value <0.05). On the right side of each plot, all module genes are shown; on the left side, transcriptional regulators (TRs) among the respective module genes are shown. Genes of interest are highlighted. **(F)** Microarray expression values of FOXP3 and MEOX1 across the CD4^+^ T cell conditions within the dataset. Conditions are colored according to the identified CCM clusters and annotated according to **Table S1**. Dashed lines indicate the computed background value of the microarray dataset.

After identifying common denominators of T cell biology, we wanted to identify the key genes specific for the respective CCM clusters. Therefore, we compared gene expression of each of the specific CCM clusters with resting T_conv_ cells. This resulted in a total of 2,385 genes which were differentially expressed (DE) (|FC| > 2, FDR adjusted p-value < 0.05) between ‘T_conv_ cell resting’ and all of the other CCM clusters in at least one condition (**Table S4**). To visualize the overlap between conditions we used an UpSet plot and observed distinct sets of DE genes for each condition but also overlap between the differentially expressed genes across conditions (**Figure 3A**). Therefore, we attempted to reduce the complexity of this analysis to identify groups of genes specific for each CCM cluster and applied Self-Organizing-Map (SOM) clustering to identify specific genes within each CCM cluster of samples (**Figure 3B**). Noteworthy, the color-coding of this clustering method revealed that every CCM cluster was characterized by a specific module-correlation structure. In addition, information about the relationships of certain CCM clusters visible in HC of CCM clusters was also preserved in SOM clustering. For example, all CCM clusters containing T_reg_ cells (‘T_reg_ cell resting’ and ‘T_reg_ cell CD3/IL-2’) exhibited high correlation with modules which were not associated with any of the other CCM clusters (**Figure 3B**). Examination of these modules unveiled T_reg_ cell specific genes such as FOXP3 but also potentially novel genes (e.g. mesenchyme homeobox 1 (MEOX1)) which have not yet been described in the context of T_reg_ cells. We also investigated modules, which were only correlated with activated CD4^+^ T cells (‘T_conv_ cell act. 8h’, ‘T_conv_ cell act. 20h’ and ‘T_H_1/T_H_2’). As expected, these modules mainly contained genes associated with T cell activation (e.g. NFKB1, INFG, TNF and ETS2).

To further investigate CCM-cluster specific gene sets, we applied weighted gene co-expression network analysis (WGCNA), which defines transcriptional modules based on the expression-correlations among genes across all samples. We identified 32 distinct modules containing 14 to 376 genes per module (**Table S5**). The expression data from all genes within a certain module were used to calculate its module eigengene (ME, the first principal component of a module), which were then correlated to the 11 clusters and visualized in a heatmap (**Figure 3C**). To identify modules containing genes which were most characteristic for a certain CCM cluster, we utilized WGCNA modules to perform gene-set enrichment analysis (GSEA). Therefore, we calculated NES, which were plotted against enrichment p-values in a Volcano plot. As representatives of the complete GSEA results, we only depicted Volcano plots for ‘T_conv_ cell act. 20h’, ‘T_conv_ cell TGF-β’, ‘T_reg_ cell CD3/IL-2’, and ‘T_reg_ cell resting’ (**Figure 3D**). As expected, among the modules with the highest positive NES and lowest p-value, we found those modules that were also most highly correlated in the WGCNA analysis to the respective CCM cluster. Next, we used the genes within the WGCNA modules with the highest ME-to-cluster correlation for further analysis. Since DE genes were utilized as input for WGCNA, we visualized the module genes by plotting the gene expression ratios between the respective cluster and ‘T_conv_ cell resting’ against p-values obtained by performing FDR-adjusted Student t-tests. The resulting Volcano plot of module genes correlated with ‘T_conv_ cell act. 20h’ revealed an enrichment of genes associated with tumor necrosis factor (TNF, LTA, TNFRSF4, TNFRSF18) and chemokine receptor ligands (CCL20, CCL4, CCL, 3, CCL3L1), whose expression is known to be increased upon T cell activation (**Figure 3E**). In addition, we found key molecules for T_reg_ cell functionality such as FOXP3, EOS, IL1R1, CTLA4, and HPGD among the WGCNA module genes correlated with ‘T_reg_ cell CD3/IL-2’. Interestingly, we also identified a transcriptional regulator, MEOX1, which was most significantly expressed in clusters containing stimulated T_reg_ cells (**Figure 3E**) and has not been associated with T_reg_ cells to date. Plotting the expression of MEOX1 across all stimulation conditions independent of CCM clustering, we identified MEOX1 expression to be highly upregulated only in condition 48, which corresponds to activated T_reg_ cells, while its expression was lower in resting T_reg_ cells (**Figure 3F**).

In summary, SOM clustering and WGCNA analysis unveiled CCM-cluster associated gene sets which were important for the biological characteristics of the respective CD4^+^ T cells. Moreover, in-depth examination of WGCNA modules associated with the cluster of ‘T_reg_ cell CD3/IL-2’ enabled us to identify MEOX1 as a novel TF associated with T_reg_ cells.

### 3.5 Further characterization of MEOX1 expression confirms its T_reg_ cell specificity

**Figure 4:**
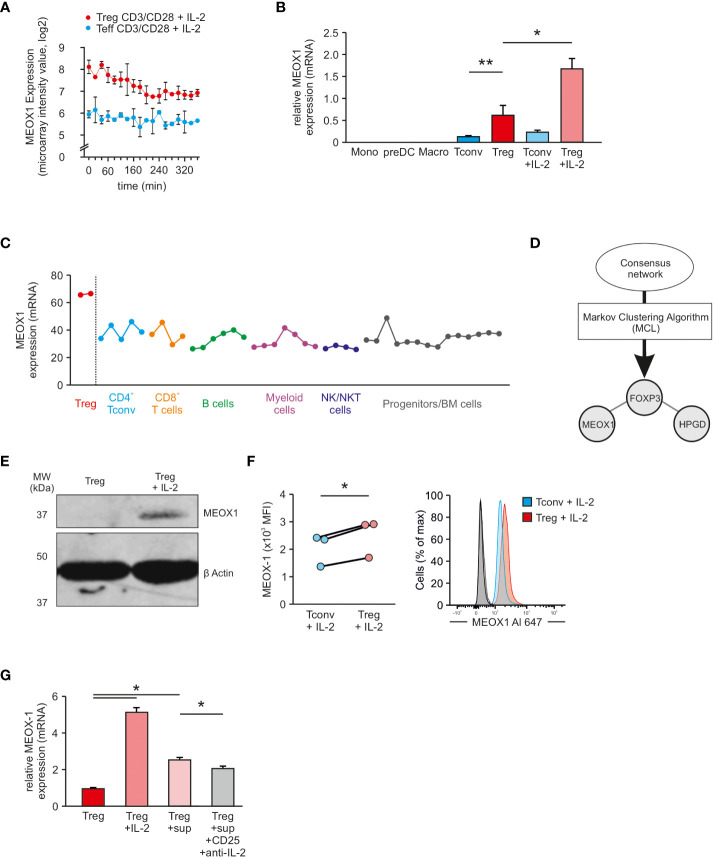
Assessment of T_reg_ cell specific expression of MEOX1 **(A)** Expression of MEOX1 in activated T_reg_ cells and T_conv_ cells over a time period of 360 min (n=2; dataset: GSE11929). **(B)** MEOX1 gene expression in different immune cells assessed by qRT-PCR and normalized to B2M (n=3). *p < 0.05 (paired Student’s t-test), ** p < 0.01 (paired Student’s t-test). **(C)** MEOX1 gene expression in different immune cells according to the NextBio database. **(D)** Application of Markov Clustering Algorithm ‘MCL’ to the consensus network generated in **Figure 2**. Visualized is a subnetwork consisting of only three genes (FOXP3, HPGD, and MEOX1). **(E)** Analysis of MEOX1 protein expression in either unstimulated T_reg_ cells or in T_reg_ cells stimulated with 100 U/ml IL-2 overnight by immunoblotting. **(F)** MFI (left) and exemplary histogram (right) of MEOX1 expression in human T_reg_ cells and naïve T_conv_ cells. PBMCs were isolated from buffy coats and stimulated overnight with 100 U/ml IL-2 (n=3 of different donors). T_reg_ cells (red) were gated on size, singlets, live, CD4^+^, CD3^+^, FOXP3^+^ (Clone 206D), CD45RA^-^ and T_conv_ cells (blue) were gated on size, singlets, live, CD4^+^, CD3^+^, FOXP3^-^ (Clone 206D), CD45RA^+^. Secondary antibody controls are depicted in light (T_reg_ cells) and dark grey (T_conv_ cells). *p < 0.05 (paired Student’s t-test). **(G)** MEOX1 gene expression in T_reg_ cells (red), T_reg_ cells stimulated with IL-2 (light ref), T_reg_ cells incubated with supernatant from stimulated T_conv_ cells (rose) and T_reg_ cells incubated with supernatant from stimulated T_conv_ cells in combination with anti-CD25 and anti-IL-2 antibodies (grey) assessed by qRT-PCR and normalized to B2M. Data is from one representative experiment of three (mean and s.e.m.) with cells derived from different donors. *p < 0.05 (two-way ANOVA).

To validate T_reg_ cell specific expression of MEOX1, we examined MEOX1-expression in an additional dataset (11). In this microarray experiment, the genome-wide expression of genes was measured by performing a high-resolution time-series analysis during the activation process of human T_reg_ cells and T_conv_ cells by treating the cells for up to 360 min with a combination of CD3/CD28-coated beads and IL-2. At each of the measured time-points, we were able to observe a higher expression of MEOX1 in T_reg_ cells than in T_conv_ cells (**Figure 4A**), similar to our own data, supporting increased MEOX1 expression in T_reg_ cells and demonstrating that the combination of CD3/CD28-coated beads and IL-2 can maintain high expression levels of MEOX1 over 6 hours.

To confirm specificity of MEOX1 expression in T_reg_ cells, we performed qRT-PCR for MEOX1 in other immune cells (**Figure 4B**). Expression of MEOX1 was solely detectable in CD4^+^ T cells, with significantly higher expression in T_reg_ cells and highest expression detectable in T_reg_ cells cultured in the presence of IL-2. We observed that the IL-2 dependent upregulation of MEOX1 mRNA is dose-independent and that a concentration of as little as 10 U/ml IL-2 is sufficient to induce a significant upregulation of MEOX1 (**Figure S1A**). This IL-2 dependent upregulation reaches its peak at around 12 hours post stimulation and then continually declines in a dose-independent manner (**Figure S1A**). This finding is in agreement with gene expression data from the public domain e.g. NextBio (**Figure 4C**) and VisuTranscript (**Figure S1B**), clearly indicating that MEOX1 is highly expressed in T_reg_ cells but not in other immune cells. Next, we asked how MEOX1 is expressed in expanded T_reg_ cells and could not observe an increase in MEOX1 expression in comparison to freshly isolated T_reg_ cells (**Figure S1C**).

Next, we reasoned that the specificity of MEOX1 expression could also be confirmed using network inference. To this end, we applied MCL (Markov Clustering Algorithm) to the consensus network obtained by the RNE and selected the generated sub-network which contained FOXP3 (**Figure 4D**). Interestingly, this network comprises only three genes: FOXP3, HPGD and MEOX1. As HPGD is also known to be important in T_reg_ cells (12), the network obtained by MCL further supported the hypothesis of T_reg_ cell specific MEOX1 expression.

Next, we validated MEOX1 expression at the protein level by western blotting. To this end, we generated a MEOX1 overexpressing vector which we transfected into HEK293 cells to show that the antibody specifically binds MEOX1 (**Figure S1D**). While we did not detect MEOX1 expression in steady-state T_reg_ cells, we observed a significant upregulation of MEOX1 in IL-2-stimulated T_reg_ cells by immunoblotting (**Figures 4E**). We confirmed these data by flow cytometry, where we showed a significant upregulation of MEOX1 in T_reg_ cells stimulated overnight with IL-2 compared with T_conv_ cells (**Figures 4F**).

Based on these results we further asked if T_conv_ cells themselves can contribute to the higher expression of MEOX1 in T_reg_ cells. To this end, we incubated T_reg_ cells with supernatants from activated T_conv_ cells and could observe increased MEOX1 expression (**Figure 4G**). To demonstrate that IL-2 is contributing to the increased MEOX1 expression, we blocked IL-2 by incubating cells with anti-IL-2Rα and anti-IL-2 antibodies and could observe a partial reduction of the increased MEOX1 expression, supporting the idea that IL-2 derived from T_conv_ cells can contribute to the increased MEOX1 expression in T_reg_ cells. Taken together, we demonstrate that MEOX1 is expressed in T_reg_ cells and upregulated in activated T_reg_ cells through IL-2.

### 3.6 Single-cell RNA-seq predicts a close co-regulation of MEOX1 and FOXP3 expression

**Figure 5:**
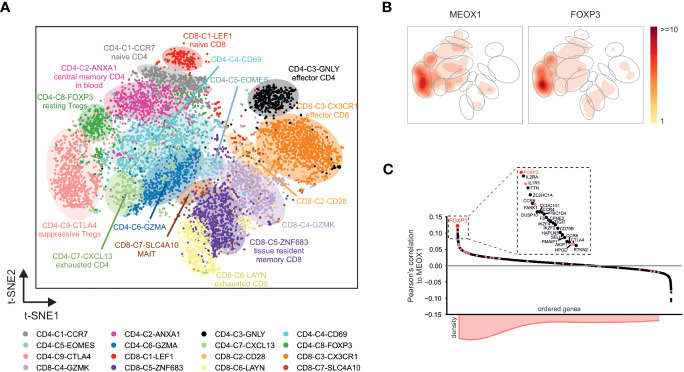
MEOX1 expression in single-cell RNA-seq data comprising different T cell populations **(A)** Visualization of single-cell RNA-seq data (GSE99254) in a t-SNE plot. Cells are colored according to the cell labels obtained by Guo et al. (28). Accumulation of cells with the same label are highlighted with colored background. **(B)** Contour plots showing the areas in the t-SNE plot of T cell clusters from GSE99254 with highest expression of FOXP3 and MEOX1. Accumulations of cells with the same cell label according to **(A)** are indicated by gray circles. **(C)** Pearson’s correlation between MEOX1 and all other genes across the CD4^+^ T cells in the single-cell RNA-seq dataset. Genes are grouped according to their respective correlation values. Genes found in the ‘T_reg_ cell CD3/IL-2’-associated WGCNA modules ‘26’ and ‘32’ (according to **Figures 3B–D**) are highlighted in light red and their accumulation in the ordered genes is indicated by the histogram at the bottom. A magnification of the 25 genes with the highest expression correlation to MEOX1 is additionally shown in the box at the top.

To investigate the expression of MEOX1 in more detail, we reanalyzed a recently published single-cell RNA-seq dataset that comprised human CD4^+^ and CD8^+^ T cell populations from non-small-cell lung cancer patients (28). After reconstruction of the t-SNE representation and the clustering of the original publication (**Figure 5A**), we visualized the expression of MEOX1. As expected, we found a trend towards higher expression of MEOX1 in the clusters “CD4-C8-FOXP3” and “CD4-C9-CTLA4” (**Figure S3A**). To validate this observation, we visualized the density of MEOX1-expressing cells in a contour plot and found the highest cell-density in the aforementioned clusters (**Figure 5B**). Interestingly, the contour plot for MEOX1 was highly reminiscent of the contour plot for FOXP3-expressing cells (**Figure 5B**), indicating a close co-expression of the two genes on single-cell level, which was also visible when plotting expression for both genes on individual cells (**Figure S3B**). Indeed, when assessing the dataset for genes correlated with MEOX1 expression, the top ranked gene is FOXP3 followed by typical T_reg_ cell-markers, such as IL2RA, IL1R1, FANK1, HPGD and CTLA4 (**Figure 5C**). Moreover, when module genes associated with the ‘T_reg_ cell CD3/IL-2’ cluster (with a FC > 2 between ‘T_reg_ cell CD3/IL-2’ and ‘T_conv_ cell resting’) were visualized in the correlation plot, we found a tendency towards positive correlation values, showing that the results obtained on bulk-scale can be reproduced on single-cell level (**Figure 5C**). In summary, the analysis of single-cell RNA-seq data substantiated the hypothesis of T_reg_ cell specific MEOX1 expression and indicated a close correlation with the expression of FOXP3.

### 3.7 Epigenetic regulation of MEOX1 expression in CD4^+^ T cells

**Figure 6:**
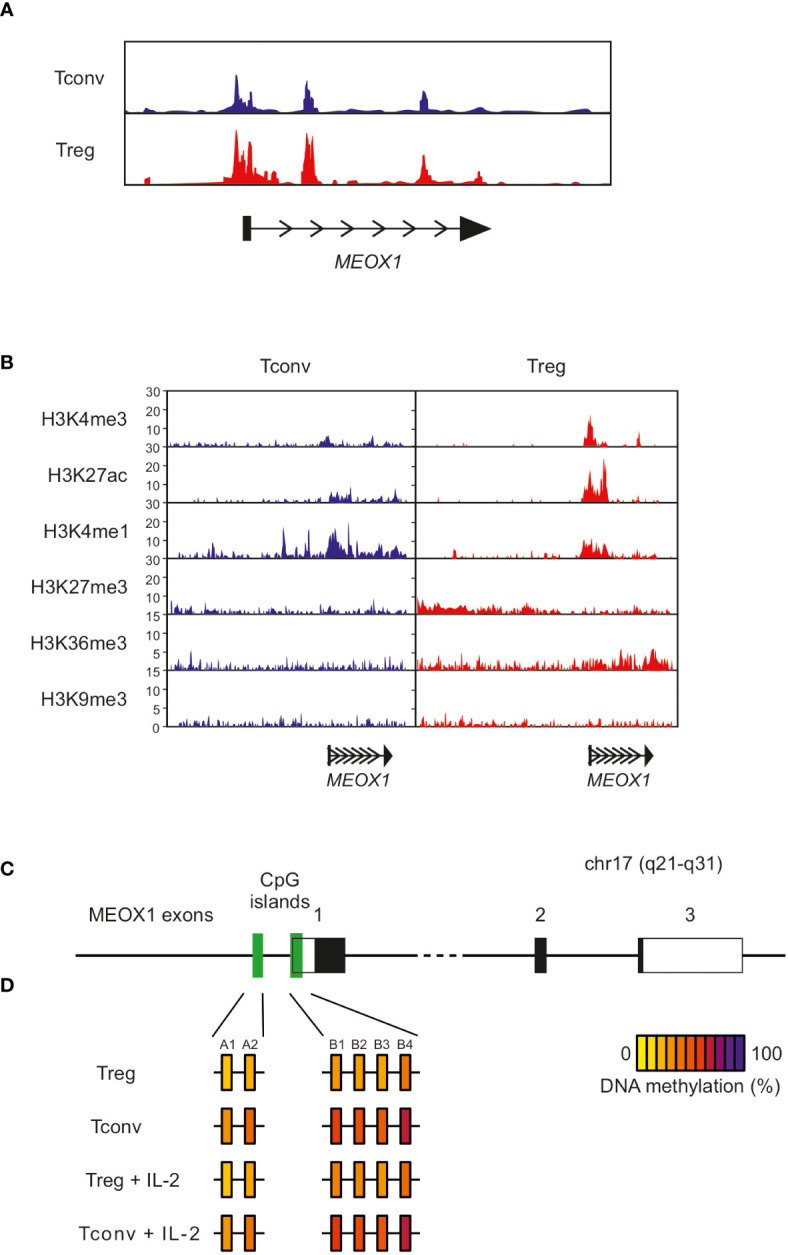
Epigenetic state of the MEOX1 gene locus in CD4^+^ T_conv_ and T_reg_ cells **(A)** Open chromatin assessment of the MEOX1 locus in T_reg_ and T_conv_ cells using ATAC-seq data. **(B)** ChIP-seq data of histone modifications at the human genomic MEOX1 locus in T_reg_ and T_conv_ cells (data obtained from the NIH Roadmap Epigenomics Mapping Consortium). Data on epigenetic regulation of the MEOX1 locus were extracted from a publicly available dataset on genome-wide histone modifications in human T_conv_ and T_reg_ cells (46). ChIP sequencing analysis of T_conv_ and T_reg_ cells for the genomic MEOX1 locus with antibodies specific for the permissive histone modifications H3K4me3 and H3K27Ac, the transcription-associated mark H3K36me1, the enhancer-associated mark H3K4me1, and the repressive histone modifications H3K27me3 and H3K9me3. **(C, D)** Methylation of individual CpG motifs within two CpG-rich regions in upstream region of the genomic MEOX1 coding sequence for freshly isolated T_conv_ and T_reg_ cell as well as T_conv_ and T_reg_ cells stimulated overnight with 100 U/ml IL-2. Each box represents an individual CpG motif after normalization and quantification of methylation signals from pyrosequencing data by calculating ratios of T and C signals at CpG sites. The methylation status of individual CpG motifs is color coded according to the degree of methylation at that site. The color code ranges from yellow (0% methylation) to violet (100% methylation) according to the color scale on the right.

One important aspect of the specification of T_reg_ cells and their transcriptional regulation are epigenetic mechanisms (63). Therefore, we determined whether differential chromatin accessibility in human T_reg_ and T_conv_ cells at the genomic MEOX1 locus contributes to the differential expression of MEOX1. Analysis of chromatin accessibility using ATAC-seq (assay for transposase-accessible chromatin using sequencing) revealed a similarly open chromatin landscape in T_reg_ and T_conv_ cells (**Figure 6A**), supporting that additional mechanisms might regulate expression of MEOX1 in T_reg_ cells. An additional layer of epigenetic regulation orchestrating cell type-specific gene expression are permissive and repressive histone modifications, particularly in close proximity to promoter regions. For example, a combination of trimethylation of H3K4 (H3K4me3) and acetylation of H3K27 (H3K27ac) indicates open chromatin states with accessible promoters and active transcription. To further extend on the epigenetic regulation of MEOX1 expression, we made use of publicly available datasets of ChIP-seq experiments which were provided by the NIH Roadmap Epigenomics Consortium (46). We observed that T_reg_ cells have a higher tag-count for both H3K4me3 and H3K27ac at the MEOX1 promoter compared with T_conv_ cells, indicating a more permissive epigenetic landscape at the promoter site in T_reg_ cells which consequently eases the transcription of this gene, as evidenced by increased H3K36me3 at the MEOX1 gene body in T_reg_ cells (**Figure 6B**). Analysis of repressive histone marks (H34K9me3 and H3K27me3) supports the notion that the activation of the transcription of MEOX1 in T_reg_ cells is an active process mediated mainly by events promoting permissive modifications of histone proteins and TF activity as we did not detect significant trimethylation of any of the two repressive marks over the gene body in T_conv_ cells (**Figure 6B**).

As DNA methylation has been reported as an additional important layer of T_reg_ cell specific gene expression, we reanalyzed publicly available datasets (64, 65) and identified two CpG islands within the promoter region of MEOX1 which were methylated in T_conv_ cells but showed significantly lower levels of methylation in T_reg_ cells (**Figure 6C**). To confirm this observation, we isolated T_reg_ and T_conv_ cells from peripheral blood, stimulated them for 24 hrs with IL-2 and assessed the methylation state of these regions by pyrosequencing (**Figures 6D**, **S4**). This analysis suggests that transcription of MEOX1 in T_reg_ cells is promoted through demethylation of the promoter region. Taken together, the epigenetic analyses of the promoter region of MEOX1 revealed an open chromatin structure with an activating histone landscape and demethylation of promoter-associated CpG islands in T_reg_ cells but not in T_conv_ cells.

### 3.8 Identification of FOXP3 as a putative upstream regulator of MEOX1 expression

**Figure 7:**
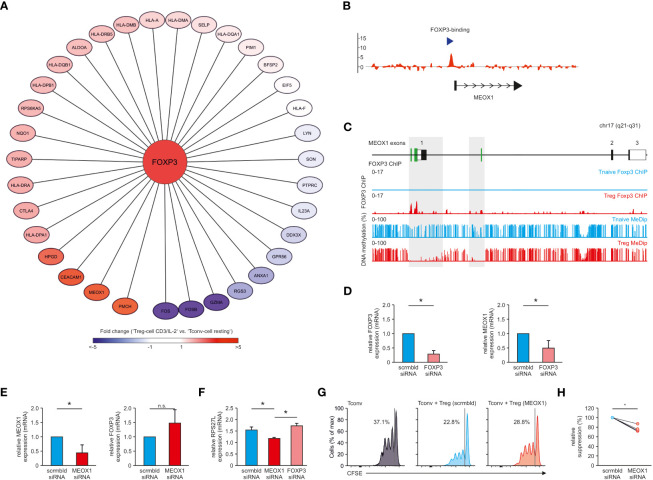
FOXP3 as upstream regulator of MEOX1 expression. **(A) **Module genes correlated with ‘T_reg_ cell CD3/IL-2’ which exhibit a FOXP3 binding-motif in their promoter region. Genes were colored according to their respective fold change (reference: ‘T_conv_ cell resting’). **(B)** FOXP3 ChIP tiling array data from human expanded cord-blood T_reg_ cells. Data were analyzed with MAT and overlayed to the MEOX1 locus to identify binding regions (p < 10^-5^ and FDR < 0.5%). Data are representative of two independent experiments with cells derived from different donors. **(C)** Overlay of MeDip-seq (66) and FOXP3 ChIP-seq data (SRA : SRP006674) for the human genomic MEOX1 locus. FOXP3 binding as well as DNA methylation is depicted for T_reg_ (red) and T_conv_ cells (blue). **(D)** mRNA expression of FOXP3 and MEOX1 in T_reg_ cells treated with scrambled (scrmbld, left) or FOXP3 specific (right) siRNA **(E)** mRNA expression of FOXP3 and MEOX1 in T_reg_ cells treated with scrambled (left) or MEOX1 specific (right) siRNA **(F)** mRNA expression of RPS27L in T_reg_ cells treated with scrambled, MEOX1 or FOXP3 specific siRNA. **(D–F)** Data were first normalized to B2M expression and shown in relation to donor-specific scrambled mRNA expression. **(D,E)** *p < 0.05 (Student’s t-test). **(F)** *p < 0.05 (two-way ANOVA). **(D–F)** Data are representative of three to five independent experiments (mean ± s.e.m.), each with cells derived from a different donor. **(G, H)** Suppression of allogeneic CD4^+^CD25^-^ T_conv_ cells labelled with the cytosolic dye CFSE by human T_reg_ cells transfected with siRNA targeting MEOX1 (MEOX1) or non-targeting siRNA (scrmbld) presented as CFSE dilution in responding T_conv_ cells cultured with CD3/CD28/anti-MHC-I antibody-coated beads and T_reg_ cells at a ratio of 1:1 **(G)**, and as relative suppression **(H)**. Data is from one representative experiment of three with cells derived from different donors. *p < 0.05 (paired Student’s t-test). n.s. = not significant.

As a next step, we investigated the transcriptional regulation of MEOX1. We postulated that potential master regulators upstream of this TF could be identified using TF-binding prediction tools based on motif-search algorithms such as iRegulon. As the input into this algorithm, we used WGCNA module-genes correlated with ‘T_reg_ cell CD3/IL-2’ cluster. This analysis identified 91 TFs with enriched binding motifs within this gene set including FOXP3 - the lineage-defining TF for T_reg_ cells (**Table S6**). We observed that several of the genes within this cluster, including MEOX1, exhibited FOXP3-binding motifs in their promoter region (**Figure 7A**), suggesting that FOXP3 is a direct regulator of MEOX1 expression. To confirm this hypothesis, we re-analyzed our ChIP-seq data for genome-wide detection of FOXP3 binding sites (10, 25), in which we detected an enrichment of FOXP3 in the promoter region of MEOX1 (**Figure 7B**).

FOXP3-mediated regulation of MEOX1 expression was further supported by re-assessing additional datasets combining DNA methylation and FOXP3 ChIP-seq data of human T_reg_ cells (27, 66). Recent work has highlighted the occurrence of hypomethylation of CpG-rich regions within FOXP3-binding regions in the genome (64, 66) and we identified MEOX1 as one of these genes showing FOXP3 binding at a hypomethylated promoter region (**Figure 7C**).

To determine whether FOXP3 is directly controlling MEOX1 expression, we performed siRNA-mediated knock-down of FOXP3 in human T_reg_ cells. This resulted in significantly reduced FOXP3 expression levels (**Figures 7D**, **S5A**) and reduced suppressive function (**Figure S5B**). When we now assessed MEOX1 expression after FOXP3 silencing, we observed significantly lower levels of MEOX1 mRNA (**Figure 7D**), supporting that MEOX1 expression is directly induced by FOXP3.

Next, we questioned whether MEOX1 also contributed to the reciprocal regulation of FOXP3 expression in T_reg_ cells. Therefore, we also silenced MEOX1 in T_reg_ cells (**Figure 7E**). While we detected a clear downregulation of MEOX1, we could not observe a direct influence of MEOX1 on FOXP3 expression (**Figure 7E**). To determine how MEOX1 influences potential downstream target genes in T_reg_ cells independent of FOXP3, we performed prediction of potential MEOX1 binding to genes specific for the cluster “T_reg_ cell CD3/IL-2”. We identified two potential MEOX1 target genes (RPL27L, PIM1) (67, 68). When we assessed their expression following knockdown of MEOX1 or FOXP3 in T_reg_ cells, we observed a MEOX1-specific upregulation of RPS27L (**Figure 7F**) and downregulation of PIM1 (**Figure S6**) independent of FOXP3, supporting the notion that MEOX1 can act as a TF supporting the transcriptional make-up of human T_reg_ cells as e.g. for PIM1 has a destabilizing function for FOXP3 as recently described (68). Considering the importance of FOXP3 for T_reg_ cell suppressive capacity, we investigated whether perturbing MEOX1 expression would also impact T_reg_ cell function. In line with our previous findings, knockdown of MEOX1 decreased the capacity of T_reg_ cells to suppress T_conv_ cell expansion *in vitro* demonstrating a functional role of MEOX1 in T_reg_ cell biology downstream of FOXP3 co-governing part of the classical T_reg_ cell properties (**Figures 7G, H**).

In summary, we used several bioinformatic approaches to identify MEOX1 as a potential novel TF important for T_reg_ cells and unveiled FOXP3 as one of the upstream regulators of MEOX1, which further corroborates the observation of a specific expression of MEOX1 in human T_reg_ cells.

## 4 Discussion

In the present study we analyzed the transcriptome of human CD4^+^ T cells by taking advantage of a highly diverse dataset. Using data dimensionality reduction methods, we observed that the highest transcriptional difference within the dataset can be attributed to the presence or absence of CD3/CD28 activation of CD4^+^ T_conv_ cells. Interestingly, these methods also revealed an unexpected close relationship between resting T_reg_ cells and resting T_conv_ cells. In several recent reports it was shown that several hundreds of transcripts are differentially expressed between T_reg_ cells and CD4^+^ T cells, by which these two cell types can be clearly defined as distinct populations at the transcriptional level (10–12, 27). Noteworthy, these reports often focused on one-to-one comparisons between resting CD4^+^ T cells and T_reg_ cells (11, 27). This approach has the tendency to overestimate the number of cell-type specific genes, as it has become evident over the last years that activation and differentiation of T cells can induce a broad spectrum of genes initially characterized as T_reg_ cell specific, e.g. CTLA-4. Hence, to our knowledge, the present study is one of the first which explicitly shows the close relationship of the transcriptional profile of human resting T_reg_ cells and resting T_conv_ cells, but also describes the distinct transcriptional modules for each subtype taking the enormous differences in gene expression into account that can be induced by T cell activation.

Global analysis of the dataset using reverse network engineering revealed CREB1, E2F3, AHR, STAT1, NFAT5 and NFATC3 as putative master regulators of CD4^+^ T cell functionality. Interestingly, we found that most of these genes were located in dense clusters of highly interconnected TFs within the consensus network. One limitation to this approach is that the importance of some of the classic T cell-related transcription factors, such as NF-κB and AP-1, as multi-protein complexes, cannot be properly captured, as their components besides FOS were not enriched in the analysis. Noteworthy, we observed several of these TF-enriched clusters which were mainly characterized by an accumulation of ZNF TFs. In regard to these observations, we postulate that the regulatory network of CD4^+^ T cells is defined by a highly interconnected TF-network consisting of a few master regulators and a plethora of adjacent ZNF TFs. This is in agreement with the observation that most of the predicted master regulators exhibit binding-motifs in their promoter region for one another, indicating a sophisticated mutual regulation of these TFs (69).

To analyze gene sets which were characteristic for clusters of transcriptionally related CD4^+^ T cells, we applied WGCNA and validated the calculated gene sets using GSEA. Besides the identification of cluster-specific transcriptional programs which have already been described to be central for T cell functionality (e.g. NFKB1 and TNF-related genes found in clusters associated with CD4^+^ T cell activation), gene set analysis led to the identification of MEOX1 in a cluster containing genes mainly expressed in T_reg_ cells stimulated with CD3 and IL-2. Next, we could show that IL-2 signaling can upregulate expression of MEOX1 in human T_reg_ cells while CD3/CD28 stimulation even in the presence of IL-2 is able to maintain the high expression of MEOX1 compared to T_conv_ cells. Our data further indicate that in humans FOXP3 is the direct transcriptional regulator of MEOX1 expression. Although FOXP3 is crucial for the suppressive function of T_reg_ cells, ectopic expression of FOXP3 in human CD4^+^ T_conv_ cells can result in induction of hypo-responsiveness and suppression of IL-2 production but might not always lead to the acquisition of suppressor activity which is characteristic for T_reg_ cells. This is in agreement with previous reports describing CD4^+^ T cells in humans which upregulate the expression of FOXP3 without the acquisition of a suppressive capacity (70, 71). These studies indicated that in humans, factors in addition to FOXP3 are required for the generation of T_reg_ cell function. To this end, we introduce MEOX1 as such a new candidate TF. The complexity of the interplay of FOXP3 with such additional factors is highlighted by the fact that expression of RPS27L as downstream target of MEOX1 is upregulated after knockdown of FOXP3 in T_reg_ cells. One further layer required for this fine-tuned transcriptional response of CD4^+^ T cells dedicated towards the T_reg_ cells fate is the respective epigenetic chromatin landscape. Here, we could show that while accessibility at the promoter region of this TF in T_conv_ cells is not different to T_reg_ cells, both histone landscape and DNA methylation pattern are distinct and might be responsible of the specific expression of MEOX1 in T_reg_ cells. In addition, this epigenetic configuration might explain why ectopic FOXP3 is not able to induce bona fide T_reg_ cells if expression of TFs like MEOX1 is prevented through epigenetic modifications.

Under the assumption that MEOX1 has an important role in regulating T_reg_ cell function, we could show that knockdown of MEOX1 in T_reg_ cells impacts T_reg_ cell suppressive activity. Furthermore, we could uncover that IL-2, potentially derived from stimulated CD4^+^ T_conv_ cells, but also other IL-2 producing cells, can upregulate MEOX-1 expression in T_reg_ cells with the contribution of other factors including costimulation still to be uncovered. With a recently published report of MEOX1 being important for the acquisition of a tumor-infiltrating T_reg_ cell phenotype (24) it is conceivable to speculate, that MEOX1 in humans is a key factor for the transcriptional acquisition of a T_reg_ cell effector phenotype downstream of IL-2 signaling. Taken together, by combining transcriptome analysis of a large dataset of T cell states with computational analysis including TF prediction, single-cell RNA-seq data of T cells, assessment of epigenetic regulation as well as *in vitro* knockdown experiments, we establish MEOX1 as a novel T_reg_ cell-specific TF. We propose that such T cell state-associated TFs and effector molecules can be identified in other modules established in this publicly available dataset of human T cell states.

## Data availability statement

The datasets presented in this study can be found in online repositories. The names of the repository/repositories and accessionnumber(s) can be found below: https://www.ncbi.nlm.nih.gov/geo/query/acc.cgi?acc=GSE15390, https://www.ncbi.nlm.nih.gov/geo/query/acc.cgi?acc=GSE17241, https://www.ncbi.nlm.nih.gov/geo/query/acc.cgi?acc=GSE20995, https://www.ncbi.nlm.nih.gov/geo/query/acc.cgi?acc=GSE11292, https://www.ncbi.nlm.nih.gov/geo/query/acc.cgi?acc=GSE99254, https://ddbj.nig.ac.jp/resource/sra-study/DRP000902, https://www.ncbi.nlm.nih.gov/geo/roadmap/epigenomics/, https://trace.ncbi.nlm.nih.gov/Traces/?view=study&acc=SRP006674.

## Ethics statement

The studies involving human participants were reviewed and approved by Ethics committee of the University of Bonn. The patients/participants provided their written informed consent to participate in this study.

## Author contributions

Conceptualization: KB, LS, LB, TU, MB. Methodology: KB, LS, MHS, TE, MK, KK, AN, SF, NO, TS. Investigation: KB, LS, MHS, TE, MK, KK, SF, NO, TS. Visualization: KB, LS, LB, TU, MB. Supervision: JH, SS, SCB, LB, TU, MB. Writing - original draft: KB, LS, LB, TU, MB. Writing - review & editing: KB, LS, MHS, TE, MK, SF, RS, NO, SS, KK, AN, JH, TS, SCB, LB, TU, MB. 
