# Supplementary material for: Identification of the novel FOXP3-dependent Treg cell transcription factor MEOX1 by high-dimensional analysis of human CD4+ T cells
Source: Front Immunol. 2023 Jul 25;14:1107397. doi: 10.3389/fimmu.2023.1107397 (PMC10407399; doi:10.3389/fimmu.2023.1107397)
Supplement: Supplementary file 1 [file DataSheet_1.pdf]

Supplementary Material

Identification of the novel FOXP3-dependent T_{reg} cell transcription factor MEOX1 by high-dimensional analysis of human $CD4^+$ T cells

Kevin Baßler[†], Lisa Schmidleithner[†], Mehrnoush Hadaddzadeh Shakiba, Tarek Elmzzahi, Maren Köhne, Stefan Floess, Rebekka Scholz, Naganari Ohkura, Timothy Sadlon, Kathrin Klee, Anna Katharina Neubauer, Shimon Sakaguchi, Simon C. Barry, Jochen Huehn, Lorenzo Bonaguro[‡], Thomas Ulas[‡], Marc Beyer[‡*]

[†] These authors contributed equally to this work and share first authorship

[‡] These authors share last authorship.

* **Correspondence:** Marc Beyer, marc.beyer@dzne.de

1 Supplementary Figures and Tables

1.1 Supplementary Figures

Figure S1

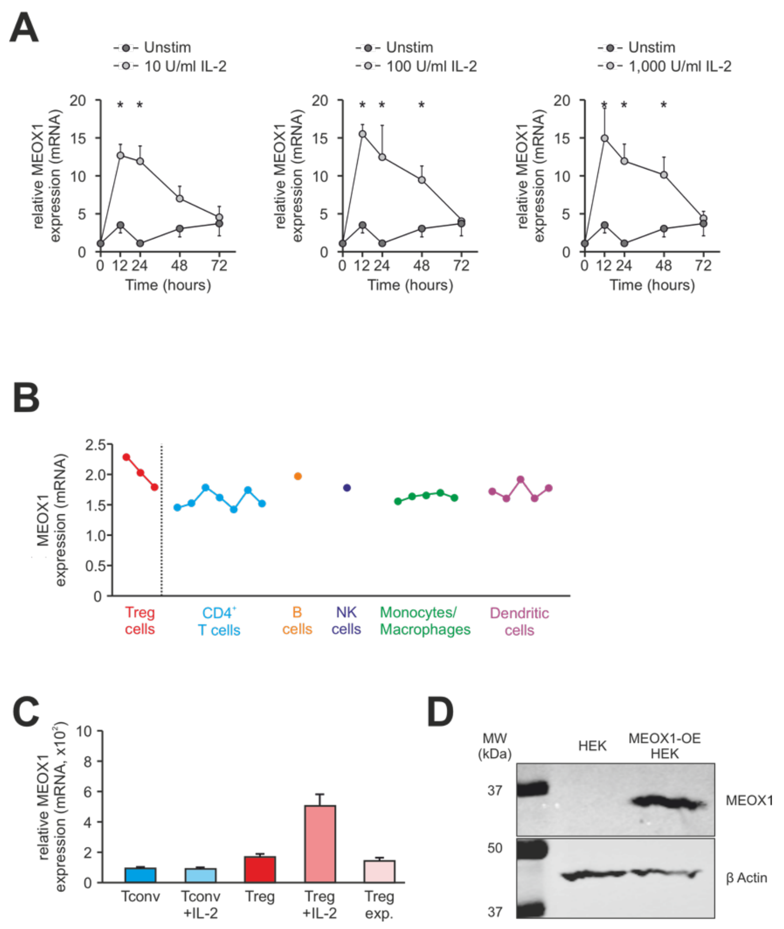

Figure S1. MEOX1 expression in T_{reg} cells.

(**A**) mRNA expression of MEOX1 in T_{reg} cells cultured for 0, 12, 24, 48 and 72 hrs in the presence of 0, 10, 100 or 1000 U/ml IL-2. *p < 0.05 (Student's *t*-test). Data are representative of three independent experiments (mean ± s.e.m), each with cells derived from a different donor. (**B**) MEOX1 gene expression in different immune cells according to the VisuTranscript database. (**C**) MEOX1 gene expression in freshly isolated T_{conv} cells, IL-2 stimulated T_{conv} cells, freshly isolated T_{reg} cells, IL-2 stimulated T_{reg} cells and expanded T_{reg} cells. (**D**) MEOX1 protein expression in non-transfected HEK cells and HEK cells overexpressing MEOX1 was determined by immunoblotting.

Figure S2

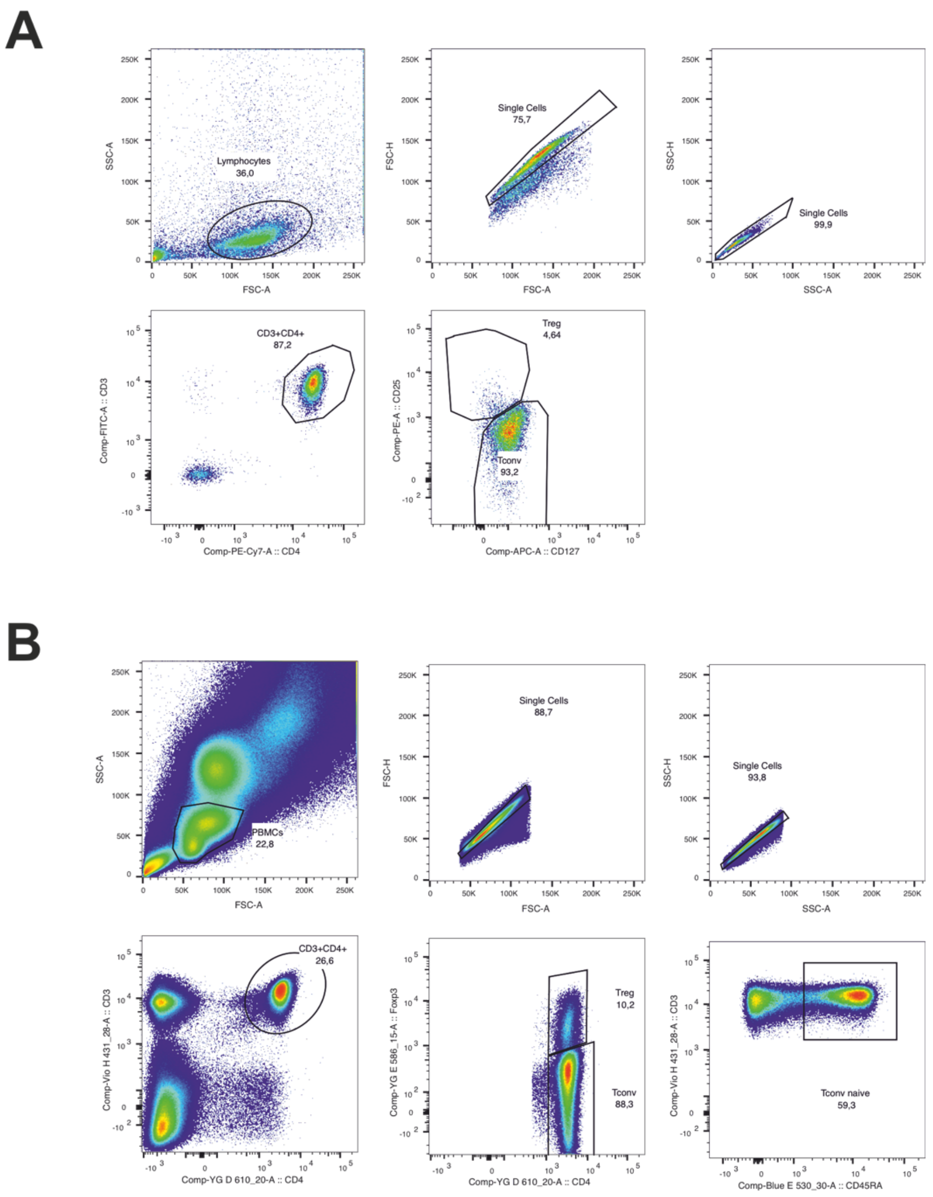

Figure S2. Gating strategy for identification and sorting of T_{reg} and T_{conv} cells.

(**A**) Gating strategy for isolation of T_{reg} and T_{conv} cells after $CD4^{+}$ T cell enrichment. (**B**) Gating strategy for identification and quantification of MEOX1 expressing T_{reg} cells and naïve T_{conv} cells.

Figure S3

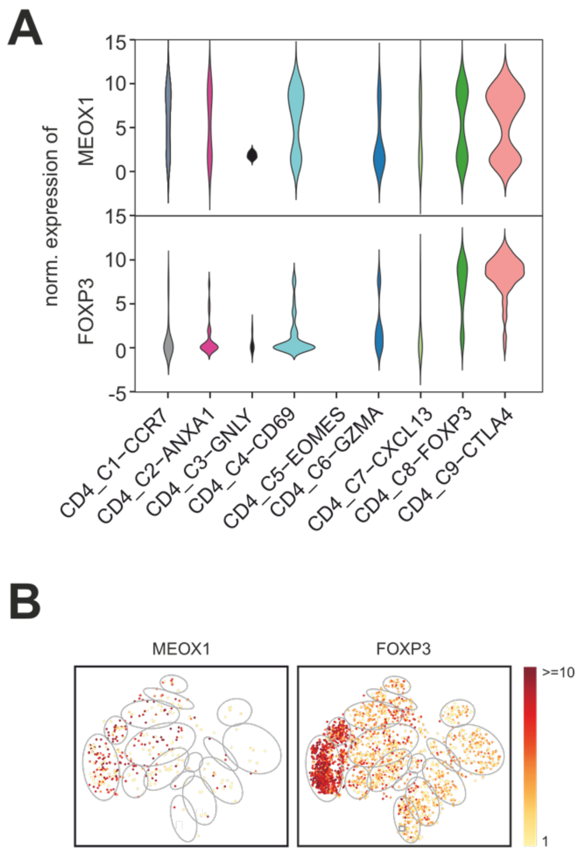

Figure S3. scRNA-seq analysis of MEOX1 expression in CD4$^+$ T cells.

(**A**) Violin plots for normalized gene expression of MEOX1 and FOXP3 in T cell clusters from GSE99254. (**B**) Expression of MEOX1 and FOXP3 were overlayed to the t-SNE plot of T cell clusters. Accumulations of cells with the same cell label according to **Figure 5** are indicated by gray circles.

Figure S4

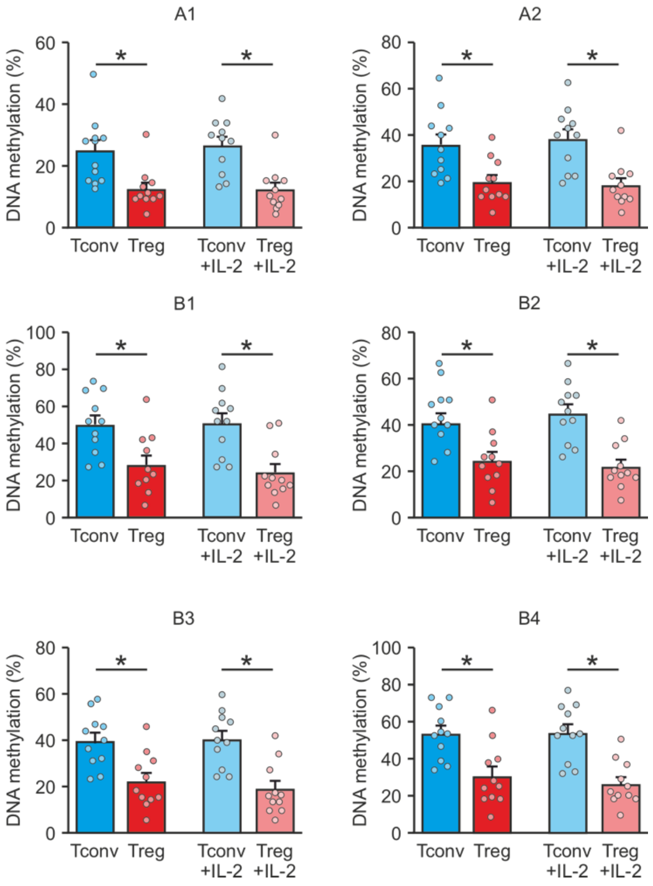

Figure S4. DNA methylation of CpG islands at the genomic *MEOX1* locus.

Methylation of individual CpG motifs within two CpG-rich regions in the upstream region of the genomic *MEOX1* locus for freshly isolated T_{conv} and T_{reg} cell as well as T_{conv} and T_{reg} cells stimulated overnight with IL-2. Individual amplicons are shown, each representing an individual CpG motif. *$p < 0.05$ (two-way ANOVA). Data are representative of two independent experiments, each with cells derived from three to five different donors (mean ± s.e.m.).

Figure S5

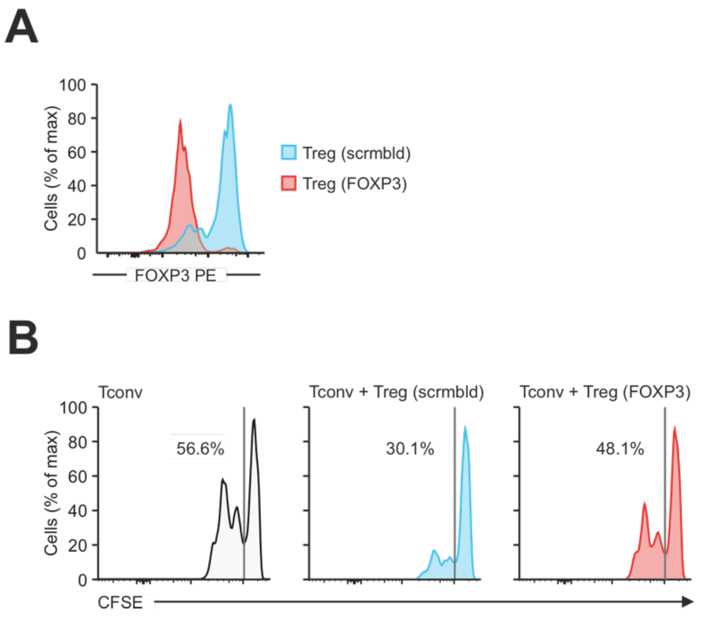

Figure S5. Knockdown of FOXP3 reduces T_{reg} cell function.

(**A**) FOXP3 expression was determined by flow cytometry (left) in human T_{reg} cells transfected with siRNA targeting FOXP3 (FOXP3 siRNA) or scrambled (control) siRNA (scrmbld siRNA) 48 hours post knockdown. (**B**) Suppression of allogeneic $CD4^+$ T cells, labelled with the cytosolic dye CFSE by human T_{reg} cells transfected with siRNA targeting FOXP3 (FOXP3) or scrambled siRNA (scrmbld), presented as CFSE dilution in responding T cells cultured with CD3/CD28-coated beads and T_{reg} cells at a ratio of 2:1 or without T_{reg} cells (T_{conv} only). (**A,B**) Data are representative of four experiments each with cells derived from a different donor.

Figure S6

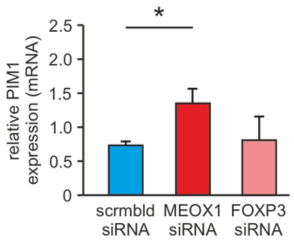

Figure S6. PIM1 expression in T_{reg} cells treated with siRNA.

PIM1 mRNA expression of T_{reg} cells treated with either scrambled, MEOX1-specific or FOXP3-specific siRNA. *$p < 0.05$ (two-way ANOVA). Data are representative of three to five independent experiments (mean ± s.e.m.), each with cells derived from a different donor.

1.2 Supplementary Tables

Table S1.
Samples and conditions used in the analysis.

Table S2.
Conditions clustered by correlation coefficient matrix (CCM) & hierarchical clustering.

Table S3.
Gene Ontology Enrichment Analysis (GOEA) for each TF together with its direct neighbors.

Table S4.
Differentially expressed (DE) genes between 'Tconv cell resting' and all of the other CCM clusters in at least one condition.

Table S5.
WGCNA based distinct modules containing 14 to 376 genes per module.

Table S6.
iRegulon analysis of TFs with enriched binding motifs within cluster 9 including FOXP3 as the lineage-defining TF for T_{reg} cells.

Table S7.
Oligonucleotides used for this study.
